# Assessment of Population Immunity Against Peste des Petits Ruminants in Goats and Sheep in India After the Second Annual Mass Vaccination Implemented Under the National PPR Eradication Programme

**DOI:** 10.3390/v18080839

**Published:** 2026-07-30

**Authors:** Kirubakaran Vinod Kumar, Rakshit Ojha, Ramachandra Deshpande, Shweta Priya, Akshatha Lokanath Goudar, Umathul Ayesha, Ponnidurai Malini, Prajakta Prashant Bokade, Anand Asha, Gangarajareddy Deekshitha, Annett Helcita Dsouza, Archana Pal, Akula Dakshitha, Shakuntala Krishnaiah Harshitha, Mahadevappa Swathi, Roopa Anandamurthy Hemanth, Lakshmipathy Archudhan, Shanmugam ChandraSekar, Kuralayanapalya Puttahonnappa Suresh, GurrappaNaidu Govindaraj, Nayakvadi Shivasharanappa, Paramanandham Krishnamoorthy, Sujit Nayak, Njeumi Felix, Baldev Raj Gulati, Satya Parida, Vinayagamurthy Balamurugan

**Affiliations:** 1 Indian Council of Agricultural Research-National Institute of Veterinary Epidemiology and Disease Informatics (ICAR-NIVEDI), Yelahanka, Bengaluru 560119, India; vinodkmr33@gmail.com (K.V.K.); ricky.ojha@gmail.com (R.O.); dramchandra643@gmail.com (R.D.); shweta2000jha@gmail.com (S.P.); akshata.goudarak004@gmail.com (A.L.G.); umathulayesha@gmail.com (U.A.); maliniponnidurai@gmail.com (P.M.); prajb95@gmail.com (P.P.B.); ashareddy457@gmail.com (A.A.); deekshitha0722@gmail.com (G.D.); annettdsouza4099@gmail.com (A.H.D.); archanapal307@gmail.com (A.P.); dakshitha.akula@gmail.com (A.D.); harshi79sk@gmail.com (S.K.H.); swathi.par10@gmail.com (M.S.); hemanthra21@gmail.com (R.A.H.); archudhanreddy@gmail.com (L.A.); suresh.kp@icar.org.in (K.P.S.); govindaraj.naidu@icar.org.in (G.G.); shivasharanappa.n@icar.org.in (N.S.); p.krishnamoorthy@icar.org.in (P.K.); baldev.gulati@icar.org.in (B.R.G.); 2 Indian Council of Agricultural Research-Indian Veterinary Research Institute (ICAR-IVRI), Mukteswar Campus, Nainital 263138, India; schand_vet@yahoo.co.in; 3 Department of Animal Husbandry & Dairying (DAHD), Krishi Bhawan, New Delhi 110001, India; sujit.nayak@nic.in; 4 Food and Agriculture Organization of United Nations (FAO), Viale delle Terme di Caracalla, 00153 Rome, Italy; felix.njeumi@fao.org (N.F.); satya.parida@fao.org (S.P.)

**Keywords:** PPR eradication, sheep and goats, mass vaccination, population immunity, epidemiological units, GCES guidelines, India

## Abstract

This study evaluated population immunity against peste des petits ruminants (PPR) in sheep and goats in India following the second annual round of mass vaccination conducted under the National PPR Eradication Programme (PPR EP), launched in 2023 in accordance with the national strategic plan. A cross-sectional post-vaccination evaluation (PVE) was conducted during August 2024 and April 2026 to assess the herd or population immunity under field conditions, in line with the WOAH and FAO Global Control and Eradication Strategy (GCES) of the PPR Global Eradication Programme (PPR GEP) 2030. A total of 52,133 serum samples were collected within 90 days post vaccination from sheep and goats across three age groups, 6–12 months (*n* = 18,002), 1–2 years (*n* = 17,316), and >2 years (*n* = 16,842), representing 1666 epidemiological units across multiple taluks and districts in 20 Indian states and Union Territories. Serum samples were tested for antibodies against PPR virus using in-house IVRI developed hemagglutinin (H) protein monoclonal antibody based competitive ELISA. The study revealed an overall population immunity of 72%, with age-wise PPRV antibody prevalence of 70.8%, 71.8%, and 72.6% in the 6–12-month, 1–2-year, and >2-year age groups, respectively. Statistical analysis showed significant associations (*p* < 0.005) between PPRV seropositivity and host factors, including species. Population immunity increased markedly from 41% (pre-vaccination sero-prevalence) in 2023 to nearly 72% by 2025-26 following two consecutive rounds of annual vaccination, accompanied by a substantial reduction in confirmed outbreaks from 57 during 2023 to 20 in 2025 in these 20 states/Union Territories. Notably, about 14.5% of epidemiological units remained below 30% seroprevalence, indicating targeted gaps requiring intensified vaccination. Overall animal vaccination coverage exceeded 95% among small ruminants aged above four months, approaching the population immunity threshold of 70 to 80%. These findings highlight the critical role of sustained successive mass vaccination campaigns in achieving and maintaining protective herd/population immunity, thereby supporting national and global PPR eradication goals.

## 1. Introduction

Peste des Petits Ruminants (PPR), commonly referred to as “small ruminant plague”, is a highly contagious viral disease that primarily affects goats and sheep, with occasional spillover infections reported in wild ruminants [1]. The disease is characterized by fever, nasal and ocular discharges, oral necrotic erosions, gastroenteritis, and bronchopneumonia [2], often resulting in high morbidity and mortality. Owing to its severe socioeconomic consequences, particularly in endemic regions, PPR represents a major constraint to small ruminant production and livelihoods. The World Organisation for Animal Health (WOAH) classifies PPR as a notifiable transboundary animal disease (TAD) [3,4]. The causative agent, PPR virus (PPRV), belongs to the species *Morbillivirus caprinae* within the genus *Morbillivirus* of the family *Paramyxoviridae*. PPR is widely prevalent across sub-Saharan Africa, the Arabian Peninsula, the Middle East, and large parts of Asia, where it continues to impose substantial economic losses [2,5,6]. In recent years, PPR outbreaks have been reported in several eastern parts of Europe, including Greece and Romania in July 2024, Bulgaria in November 2024, Hungary in January 2025, Albania in June 2025, Kosovo in July 2025, Croatia in December 2025, and additional outbreaks in early 2026 [7]. PPRV lineage IV was also detected for the first time in Vietnam in October 2025 [8]. These events indicate the continued geographic expansion nature of PPR within the European countries [9] and transboundary spread of PPR, highlighting the need for sustained global surveillance and control measures [7,8].

To address the global threat posed by PPR, international initiatives such as the PPR Global Eradication Programme (PPR-GEP) and the Global Control and Eradication Strategy (GCES) were launched by WOAH and FAO, with the objective of achieving worldwide eradication [10], and both the FAO and WOAH have initiated the second phase of the PPR-GEP in 2022 and unveiled a blueprint for recommended activities covering 2022–2030, aiming to achieve global PPR eradication by 2030 [11]. Effective control of PPR relies on the availability of safe and efficacious live attenuated vaccines, reliable diagnostic tools for rapid detection, robust surveillance systems, and the strategic implementation of timely vaccination campaigns. Vaccination remains the cornerstone of PPR prevention and control. Currently, two live attenuated vaccines, Nigeria 75/1 and Sungri/96, developed using Vero cell culture techniques, are widely produced and used across endemic regions. These vaccines, endorsed under the PPR-GEP, have proven instrumental in reducing disease incidence.

In India, where PPR is endemic, control efforts were initiated well before the establishment of the global PPR-GEP. Vaccination activities began in 2006 through targeted outbreak-focused immunization to limit virus spread. Subsequently, in 2011, the Department of Animal Husbandry and Dairying (DAHD), Government of India (GoI), launched the national PPR Control Programme (PPR-CP), emphasizing mass vaccination, rapid diagnosis, surveillance, monitoring, and disease prevention. Since the implementation of strategic mass vaccination, reported PPR outbreaks in India have declined steadily, accompanied by noticeable changes in disease patterns, severity, and geographical distribution [2]. These improvements highlight the effectiveness of vaccination and programmatic interventions in sheep and goat populations.

Building on these gains, India launched the PPR Eradication Programme (PPR-EP) during 2022–2023 under the Critical Animal Disease Control Programme (CADCP) of the Livestock Health Disease Control Programme (LHDCP), following extensive stakeholder consultations and in alignment with the GCES framework [3]. The PPR vaccination campaign in India is a key component of the National Strategic Plan (NSP) for PPR Eradication 2030, implemented by the DAHD in coordination with State Animal Husbandry Departments. It aims at progressive interruption of virus transmission and eventual eradication through sustained vaccination and herd immunity in sheep and goat populations [3]. The national strategic plan emphasizes mass vaccination of high-risk populations, including migratory flocks and border regions, targeting > 90% animal vaccination coverage and achieving over 80% herd immunity. The strategy targets blanket vaccination up to 2027 through pulse vaccination (animals above 4 months of age) over 3–4 rounds of annual mass vaccinations to achieve 70–80% herd immunity, followed by immunity assessment and intensified surveillance to interrupt transmission by 2027, cessation of circulation by 2028, and PPR-free status by 2029–2030. The surveillance framework includes sero-monitoring, outbreak investigation, syndromic and sentinel surveillance, including livestock–wildlife interface monitoring. ICAR-Indian Veterinary Research Institute (IVRI) and ICAR-National Institute of Veterinary Epidemiology and Disease Informatics (NIVEDI) serve as national reference centres for diagnosis, sero-surveillance, epidemiology, data management, and capacity building, supported by a national laboratory network. The programme also integrates vaccine quality control, real-time vaccination monitoring, cold-chain logistics, and training of field personnel for effective implementation [12].

Despite significant progress, sporadic PPR outbreaks continue to be reported in certain regions of India, largely attributed to gaps in surveillance and vaccination implementation challenges [3]. Strengthening governance, veterinary service delivery, and surveillance systems by drawing lessons from the rinderpest eradication experience remains critical for achieving PPR eradication. In this context, ICAR-NIVEDI in collaboration with DAHD, GoI and ICAR-IVRI initiated a national action plan for PPR surveillance and monitoring under the PPR-EP. A nationwide cross-sectional population immunity study was undertaken between August 2024 and April 2026 using a multistage stratified random sampling approach in accordance with PVE protocol I WOAH and FAO guidelines.

Findings from the nation-wide pre-vaccination study conducted during 2023–2025 in different states/Union Territories at various periods indicate an overall seroprevalence of approximately 40.53%, with state-wise prevalence ranging from 1.4% (Nagaland) to 82.13% (Telangana) across 33 states/UTs covering 3099 villages, using a huge number of 83,196 serum samples (unpublished data). Importantly, more than 1500 epidemiological units reported seroprevalence below 30%, highlighting critical immunity gaps and the urgent need for intensified vaccination efforts. These findings provide valuable baseline data to guide vaccination strategies and support India’s contribution to the global PPR eradication initiative.

Currently, in India, only the Sungri/96 PPR vaccine strain is commercially available, and this live attenuated vaccine provides long-term protective immunity (over 3 to 6 years) against circulating PPRV [3]. Achieving 70–80% herd or population immunity through annual mass vaccination coverage of animals, along with the efficacy and effectiveness of the vaccine in sheep and goats under real-world field conditions, is critical to disrupting the PPRV epidemic cycle. Despite vaccination efforts in India, PPR outbreaks are still sporadically reported in parts of the country, mainly due to bottlenecks in countrywide vaccination and surveillance [2,13,14]. Prevalence studies to assess PPRV antibody prevalence and population immunity status are crucial for PPR eradication, including PVE or sero-monitoring to determine vaccine efficacy and vaccination effectiveness. In this context, the present study assesses population immunity status against PPR in sheep and goats in India following the second annual round of mass vaccination implemented under the National PPR EP, launched in 2023 in accordance with the national strategic plan.

## 2. Materials and Methods

### 2.1. Study Area

The study was conducted across 20 Indian states and union territories, from among the 28 States and 8 Union Territories (UTs) that had completed the Second Round of Mass Annual Vaccination under the PPR-EP of the NSP 2030, representing diverse agro-climatic zones and small ruminant production systems, namely Andhra Pradesh, Assam, Bihar, Chandigarh, Chhattisgarh, Goa, Gujarat, Karnataka, Ladakh, Madhya Pradesh, Maharashtra, Meghalaya, Mizoram, Odisha, Puducherry, Punjab, Tamil Nadu, Telangana, Uttarakhand and West Bengal (Table 1). These states encompass varied geographical landscapes, ranging from coastal plains to plateaus and hilly regions, and reflect heterogeneity in livestock density and animal husbandry practices.

All selected states were early adopters of the PPR-EP and implemented mass vaccination under the NSP during 2024–2026. All studied states completed at least the second round of annual vaccination and met the eligibility criteria for systematic PVE in accordance with GCES guidelines.

**Figure 1:**
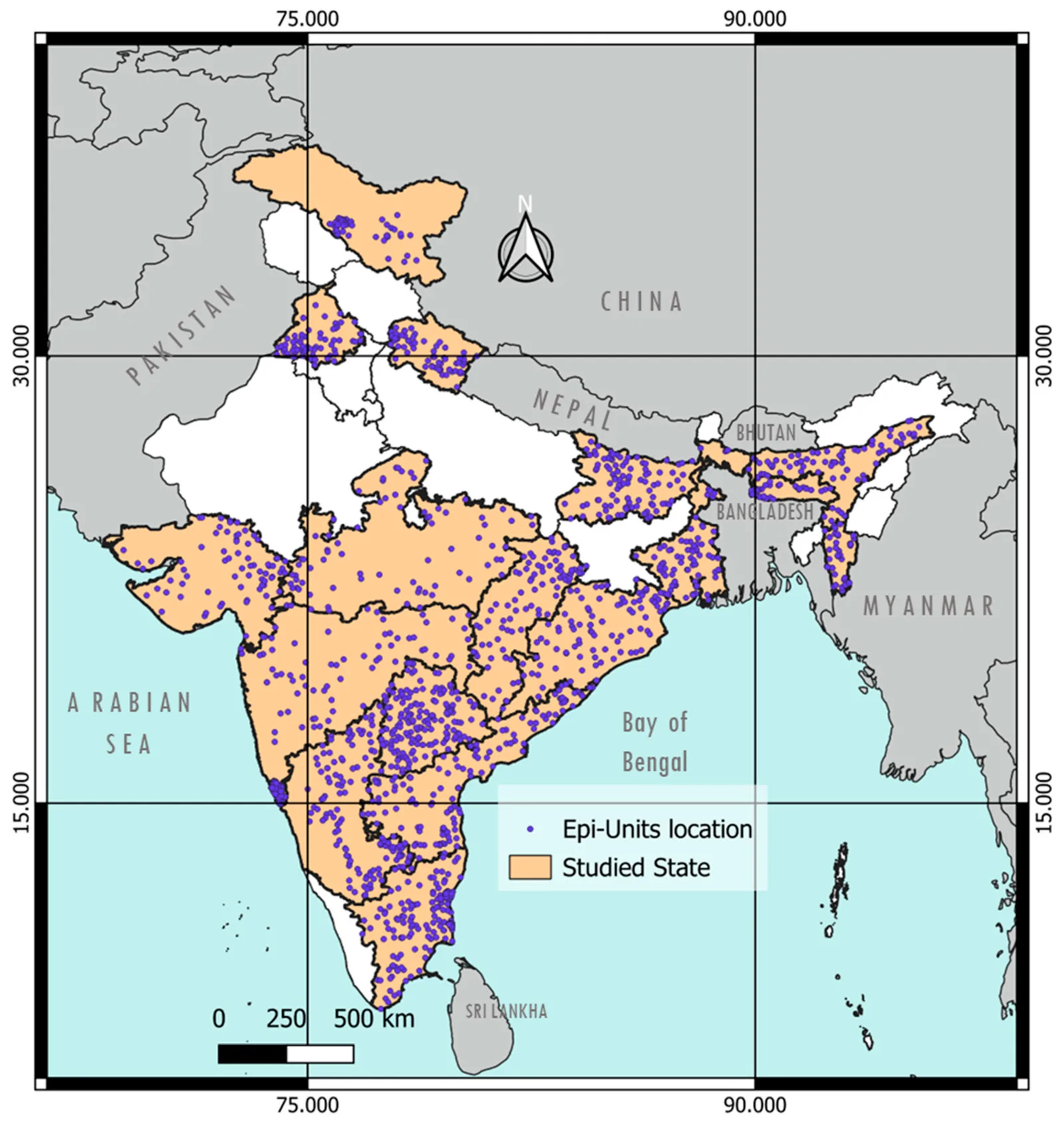
Survey for population immunity status of PPR in Goats and Sheep in India after the Second Annual Mass Vaccination implemented under the National PPR Eradication Programme of the Livestock Health and Disease Control Programme (LHDCP), Government of India. The locations of surveyed epidemiological units (villages) across 20 States/UTs from which serum samples were collected are depicted as filled dots on the GIS map of India.

According to the 20th Livestock Census of India (2019), the study states together account for approximately 60.95 million sheep and 99.18 million goats, totaling 160.13 million small ruminants across the 20 surveyed states (Table S1; Department of Animal Husbandry and Dairying, Government of India; accessed 5 January 2026). The spatial distribution of surveyed epidemiological units (epi-units) is presented in Figure 1, demonstrating comprehensive geographic coverage.

### 2.2. Study/Target Population

The target population comprised susceptible sheep and goats in the selected states. A village was defined as an epidemiological unit (epi-unit) based on its distinct socio-economic characteristics and animal husbandry practices. Animals within each epi-unit were stratified into three age groups: 6–12 months, 1–2 years, and >2 years. These age categories were used for serological assessment of population immunity and evaluation of vaccine effectiveness.

### 2.3. Study Design and Sampling Strategy

A cross-sectional study design was employed to estimate the prevalence of PPRV antibodies in sheep and goats following the second round of mass vaccination (2024–2025) under the PPR-EP. Sampling procedures followed the WOAH/FAO GCES-PVE Protocol I [10,15]. A two-stage stratified random sampling design was adopted. Stage 1: Epi-units (villages) were randomly selected from each state at different districts, using an inclusion criterion of >500 small ruminants or 50–100 animals per village based on population density in each state. Randomization was performed in levels using the NIVEDI Epi-Calculator software (Version 2.0) (accessed 5 January 2023) [15]. Stage 2: Within each selected epi-unit, three to four households/flocks were randomly chosen, each maintaining a minimum of 20 animals per flock. Animal-level sampling within households was performed using convenience sampling, as recommended in the GCES protocol.

### 2.4. Sample Size Estimation

Sample size estimation was conducted following WOAH/FAO GCES guidelines [10,15]. For pre-vaccination surveys, an expected prevalence of 30% was assumed. For post-vaccination sero-monitoring, 70% immune coverage was assumed in states lacking prior immunity data. Diagnostic test parameters, including 92.4% sensitivity and 98.4% specificity of the PPR competitive ELISA (c-ELISA), were incorporated [16]. The target system sensitivity was set at 95%. Based on these assumptions, the required number of epi-units was estimated at the state level. Within each epi-unit, animal-level sample sizes were calculated using a hypergeometric distribution, assuming a 30% animal-level prevalence. As most epi-units had populations exceeding 94 animals, 9–10 animals per age group were sampled. Post-vaccination sampling was conducted 60–90 days after vaccination to allow sufficient time for seroconversion (minimum 21 to 28 days).

#### Statistical Basis for Epi-Unit Sample Size Calculation

Epi-unit sample sizes were calculated using the following inputs: animal-level prevalence of non-protection (1 − P) derived from the previous year’s first round of annual mass vaccination sero-monitoring results; cluster-level prevalence, estimated using state-wise small ruminant population density (total population/geographical area), stratified into four density classes: 0–30 (16%), 30–60 (14%), 60–100 (12%), and >100 (10%); diagnostic sensitivity (92.4%) and specificity (98.4%) of the In-house IVRI-developed PPR c-ELISA; and baseline immunity levels from earlier monitoring rounds. A two-stage stratified random sampling design was generated at a 95% confidence level with 5% allowable error using an in-house epi-calculator. State-wise epi-unit sample sizes and corresponding Data Requirement Specifications (DRS) are presented in Table 1.

### 2.5. Population Immunity Assessment

Population immunity was assessed as a proxy measure of vaccination campaign effectiveness using post-vaccination serological surveys. Sampling was conducted 30–90 days after vaccination. It was assumed that at least 50% of vaccinated epi-units would achieve ≥ 70% seropositivity in the 6–12-month age group, which served as the primary indicator of recent vaccination efforts. Population immunity was estimated as the proportion of epi-units achieving ≥ 70% protection among all aged strata of animals and compared with corresponding pre-vaccination baseline seroprevalence in all age groups for vaccination impact. Within each selected flock, a maximum of 3–4 animals per age group were sampled (6–12 months, 1–2 years, and >2 years). The state-wise sampling framework for PPR-PVE of sero-monitoring under Sampling Plan 3 (SP3) is summarized in Table 2.

Participating States were identified by the respective State Animal Husbandry Departments. Within each State, epidemiological units (villages) were selected by simple random sampling from a village sampling frame prepared by ICAR–NIVEDI and shared with State authorities. Within selected villages, households or flocks were randomly chosen using locally available livestock records and field veterinarian. At the animal level, sampling within flocks was based on the availability of eligible animals in predefined age groups, as complete animal lists were generally not available and restraining all eligible animals was not feasible under field conditions. This resulted in convenience-based selection restricted to animals present at the time of visit and meeting programme-defined inclusion criteria. If a selected flock was inaccessible or unavailable, another eligible flock within the same epidemiological unit was substituted to maintain the required sample size, as per GCES guidelines [3,14].

### 2.6. Study Design for Pre-Vaccination Status

To enable direct comparison with the pre-vaccination immunity status, the post-vaccination seromonitoring survey followed the same study design, sampling framework, laboratory methods, and analytical procedures as the nationwide baseline serosurvey conducted by ICAR–NIVEDI during 2023–2024, immediately before implementation of the National PPR-EP [15]. The baseline survey covered 33 States/UTs and was conducted in accordance with WOAH/FAO GCES Strategic PVM Protocol 1 [10]. A multistage stratified random sampling design was adopted, with villages serving as epi-units. Villages having > 500 or >100 small ruminants, as identified from the 20th Livestock Census (2019) of each state, were randomly selected using the NIVEDI Epi-Calculator [3]. A total of 108–120 villages per State/UTs were included based on the consideration of sensitivity of the assay (PPR c-ELISA) employed for screening, and 3–4 flocks per village were randomly sampled. Approximately 27–30 sheep and goats per village (9–10 animals each from the 6–12 months, 12–24 months, and >2 years age groups) were sampled, yielding a minimum of 3240 serum samples per State/UTs. The post-vaccination survey used after the second round of mass vaccination was employed in the present study, including the epidemiological unit-based sampling strategy, age stratification, sample size, and laboratory procedures, ensuring valid comparison of seroprevalence estimates before and after implementation of the National PPR EP.

### 2.7. Sample Testing and Analysis

During PVE surveys conducted between 2024 and 2026, a maximum of 30 serum samples per epi-unit were collected, with no more than 10 samples per age group. From each household/flock, 3–4 animals per age group were sampled, maintaining predefined species and population proportions in accordance with WOAH guidelines [15]. In epi-units where only sheep or goats were present, samples were collected from the available species only. Sampling was carried out with the support of the State Department of Animal Husbandry and Veterinary Services. Animal-level data, including species, age, sex, and breed, were recorded using a structured questionnaire. Geographic coordinates of sampled villages were mapped using QGIS software (version 2.18.6) and are shown in Figure 1.

Serum samples were transported to the WOAH Reference Laboratory for PPR (ISO/IEC 17025:2017 accredited) at ICAR–NIVEDI, Bengaluru, and stored at −20 °C until analysis. All serum samples were tested using In-house IVRI-developed PPR c-ELISA kit [17], which uses H protein specific mono monoclonal antibody and detects antibodies against the hemagglutinin (H) protein of PPR virus. Results were expressed as percentage inhibition (PI), and samples with PI ≥ 50% were considered as a clear seropositive and protective antibody response against PPR vaccine immunization. Data from all participating States and Union Territories were compiled, cleaned, validated, and tabulated using Microsoft Excel 2019. All statistical analyses were performed using R statistical software (version 2025.05.1). Seroprevalence was calculated as the proportion of c-ELISA-seropositive animals among those tested and expressed as percentages with 95% confidence intervals, where appropriate. Associations between seropositivity and host factors (species, age, sex, breed, and State/Union Territory) were assessed using Pearson’s chi-square test or Fisher’s exact test, as appropriate [18]. Statistical significance was set at *p* < 0.05.

## 3. Results

**Figure 2:**
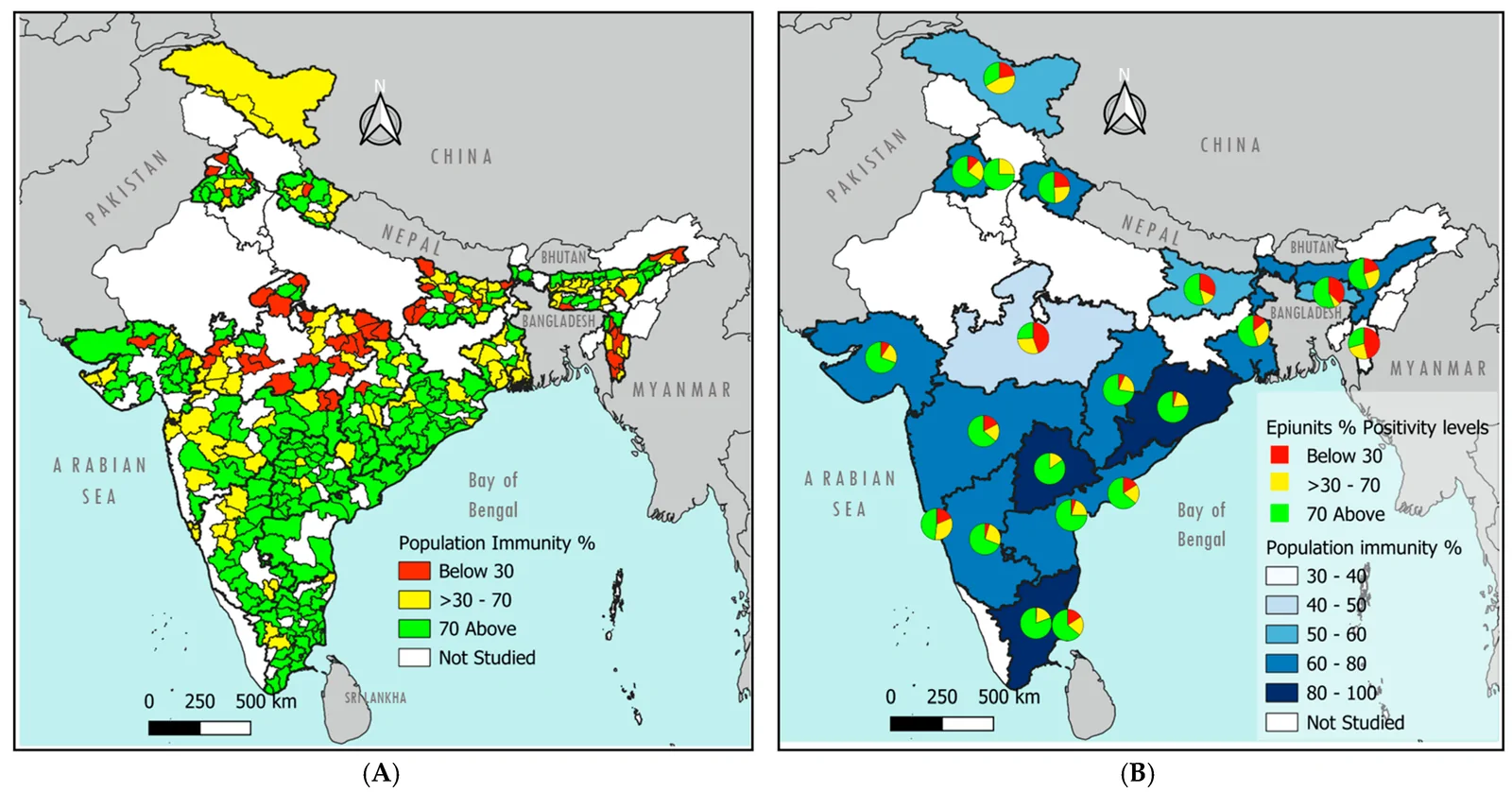
(**A**) District-wise distribution of PPR virus antibody prevalence (population Immunity % seroprevalence) in small ruminants across 20 States/UTs of India, covering multiple epidemiological units. Districts shown in white indicate areas where no sampling was conducted in this study. (**B**) State-wise population immunity status for PPR (% seroprevalence) in small ruminants across 20 States/UTs of India. Shaded gradients represent state-level population immunity (%) based on seroprevalence, while pie charts illustrate the distribution of epidemiological units (epi-units) by seropositivity levels: red for below 30%, yellow for 30–70%, and green for above 70%. The map depicts the immunity status of PPR in Goats and Sheep in India after the Second Annual Mass Vaccination implemented under the National PPR Eradication Programme of the Livestock Health and Disease Control Programme (LHDCP), Government of India.

The overall seroprevalence of PPRV antibodies across the surveyed epidemiological units (epi-units) in India under the PPR-EP was 72%, reflecting the immunity status of small ruminants following two rounds of mass vaccination (Table 1). Species-wise analysis revealed a significantly higher prevalence in sheep (78.2%) compared to goats (69.3%). A state-wise summary of sera tested, PPRV antibody positivity, and corresponding population immunity is presented in Table 1 and Figure 2B, while the district-wise distribution of seroprevalence is illustrated in Figure 2A. Among the states, Telangana exhibited the highest population immunity (85.4%), whereas Mizoram recorded the lowest 39.8% immunity. Age-wise analysis indicated comparable levels of seropositivity across age groups: 70.8% in animals aged 6–12 months, 71.8% in 12–24 months, and 72.6% in animals older than 2 years of age.

**Figure 3:**
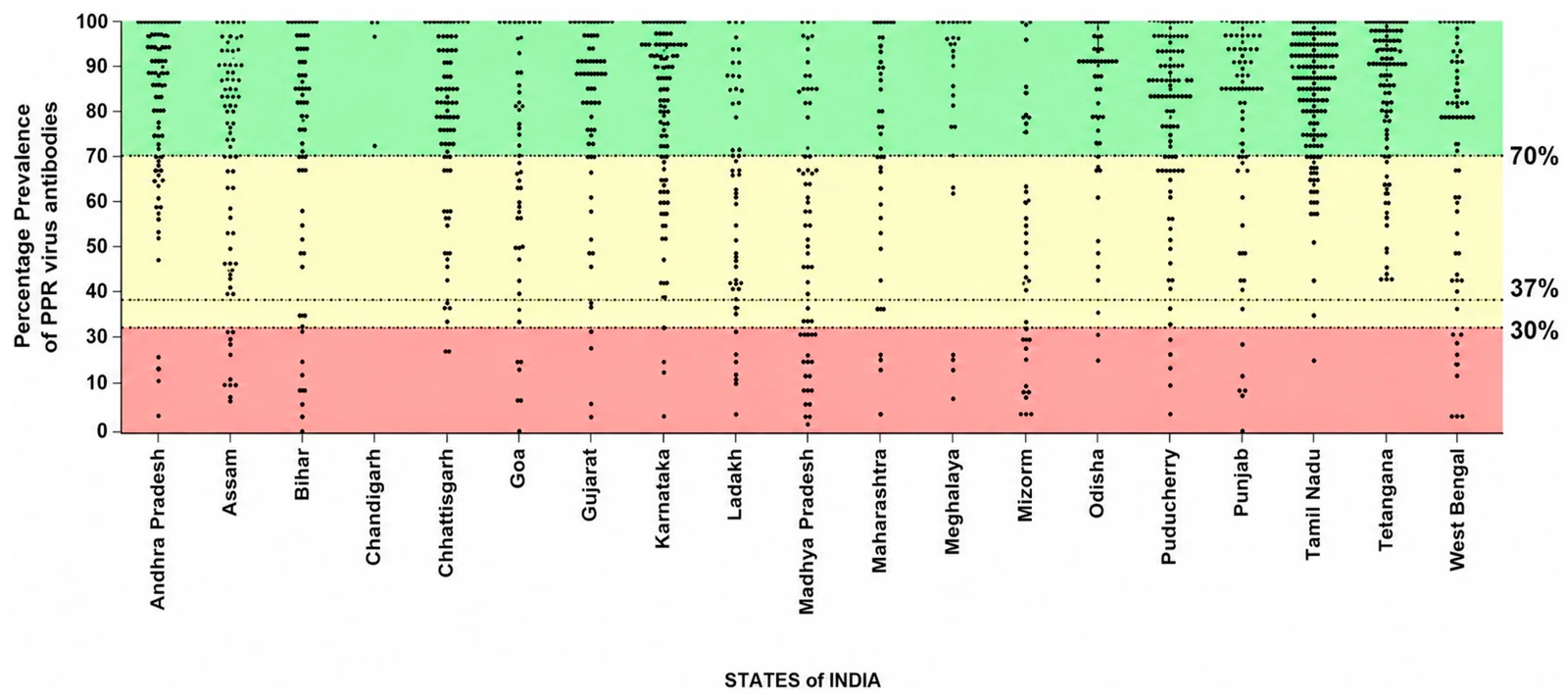
Distribution of PPR virus antibody prevalence (population Immunity % seroprevalence) in small ruminants across 20 States/UTs of India, covering multiple epidemiological units. These sero-monitoring results for PPRV antibodies in small ruminants across surveyed states show the immunity status at the state/UT level, highlighting the percentage prevalence of antibodies in small ruminants after the Second Annual Mass Vaccination.

Statistical analysis showed significant associations (*p* < 0.05) between PPRV seropositivity and host factors. The Chi-square analysis revealed significant differences in PPRV antibody prevalence between species (χ^2^ = 410.24, *p* < 0.05) and substantial inter-state variation in seroprevalence for both sheep and goats (χ^2^ = 3758.9, *p* < 0.05). Significant associations (*p* < 0.05) were observed between PPRV seropositivity and host-related factors including age group (χ^2^ = 13.71), sex (χ^2^ = 113.3), and animal husbandry practices (χ^2^ = 481.8), whereas no significant association was found with breed (χ^2^ = 7.57, *p* > 0.05) (Table S23). The distribution of PPRV antibodies across states/UTs and epi-units is shown in Figure 3 and Table 1 and Table S22. At the epi-unit level, substantial heterogeneity in immunity was observed. Of 1666 epi-units surveyed, 63.3% (*n =* 1054) exhibited high seroprevalence (>70%), 14.5% (*n =* 242) showed low seroprevalence (≤30%), and 22.2% (*n =* 370) had intermediate seroprevalence (31–70%). These results indicate persistent immunity buildup over just two rounds of annual mass vaccination, underscoring the need for zone-wise evaluation to guide targeted vaccination strategies to achieve compartmentalization or zone-wise PPR eradication strategies in the country, as some of the states of India entered stage 3 as per PPR Monitoring and Assessment Tool (PMAT) evaluation. Baseline PPRV antibody seroprevalence prior to mass vaccination under India’s PPR-EP showed marked spatial heterogeneity across the geographical zones of India. These patterns reflect differences in historical vaccination coverage, livestock management practices, accessibility, and programmatic implementation. Overall, the population immunity patterns demonstrate that southern and parts of western India have achieved substantial immunity, whereas other regions continue to exhibit significant immunity gaps (Table S22). With 14.5% of epi-units nationwide showing < 30% seroprevalence, the findings strongly support the need for high-coverage (>95%) mass vaccination, reinforced surveillance, and post-vaccination evaluation under the NSP for PPR Eradication to achieve sustained PPR-free status in India.

## 4. Discussion

Population immunity trend assessment is a cornerstone for the successful eradication of PPR, as emphasized in the WOAH/FAO guidelines, which recommend achieving herd immunity above 70–80% to interrupt virus transmission and sustain PPR-free status [10,15]. Monitoring PPRV antibody prevalence in small ruminant populations is therefore essential for evaluating the effectiveness of mass vaccination campaigns and guiding strategic interventions under national PPR EP [10].

In the present study, the overall seroprevalence of PPRV antibodies across surveyed epidemiological units in India was 72% following two rounds of mass vaccination, indicating moderate-to-high immunity in the small ruminant population. Sheep showed significantly higher seroprevalence (78.2%) than goats (69.2%), consistent with previous reports suggesting species-specific differences in vaccine response and longer persistence of antibodies in sheep compared to goats, which are often subjected to early-age marketing [3]. Age-wise analysis demonstrated comparable seropositivity across age groups, including younger animals (6–12 months, 70.8%), suggesting partial immunity development even among cohorts born after the first vaccination round [3]. Considerable spatial heterogeneity in immunity was observed at both the state and epi-unit levels. While most states achieved seroprevalence above 70%, a few remained below 60%, with Mizoram recording the lowest immunity (<40%). In contrast, Telangana exhibited the highest population immunity (>90%), demonstrating that well-implemented vaccination programmes can achieve immunity levels sufficient to substantially reduce virus circulation and support PPR eradication efforts. Such heterogeneity likely reflects differences in vaccination coverage, implementation efficiency, accessibility, livestock demographics, and local epidemiological conditions. At the epi-unit level, only 14.5% of units showed low seroprevalence (<30%), whereas the majority (63.3%) exhibited high seroprevalence (>70%), highlighting the persistence of immunity after annual mass vaccination rounds.

Despite completion of the second vaccination round, population immunity remained suboptimal in some states, suggesting that reported vaccination coverage exceeding 70% may not necessarily translate into adequate herd immunity. Several factors may contribute to these gaps, including logistical constraints, difficult terrain, limited access to remote livestock populations, rapid turnover of small-ruminant populations, and continuous introduction of susceptible animals through births, deaths, sales, and animal movement. In addition, young animals born after vaccination campaigns become increasingly susceptible as maternal antibodies wane [19]. Variations in vaccine delivery, cold-chain maintenance, farmer participation, and timing of post-vaccination sampling may also influence observed seroprevalence estimates. Similar vulnerability was observed in Bihar and parts of the North East regions, where seroprevalence remained below 60%, possibly due to gaps in vaccination coverage, difficult geographical access, and high animal movement compared to southern states. These findings highlight the need for intensified vaccination, continuous seromonitoring, and targeted corrective vaccination to improve herd immunity in low-immunity regions. Risk-based interventions focusing on high-risk regions, border areas, and trade/transit points may further enhance immunity and prevent virus reintroduction. Periodic serological surveillance should complement administrative vaccination coverage data to provide a more accurate assessment of population immunity and identify residual immunity gaps requiring corrective interventions.

Moreover, a nationwide baseline serosurvey conducted using the same WOAH/FAO GCES protocol and c-ELISA methodology included 83,196 samples from 3099 epidemiological units across 33 States/UTs and reported an overall seroprevalence of 41%, with over 1500 epidemiological units showing seroprevalence below 30%, indicating substantial immunity gaps before mass vaccination (unpublished data). Although both surveys were independent cross-sectional studies, the identical methodology permits programme-level comparison, with the present study demonstrating an increase in seroprevalence to ≈72% following implementation of the National PPR EP. This should be interpreted in the context of the nationwide baseline serosurvey conducted by ICAR-NIVEDI using the same WOAH/FAO GCES sampling protocol, sample size estimation, age stratification, and c-ELISA methodology. Similarly, the nationwide PPR serosurvey conducted during 2017–2018 revealed low overall immunity (43.68%) with significant regional variation, highlighting critical gaps in protective coverage [13,16,20,21,22,23,24]. However, the persistence of numerous low-immunity epi-units also reflected the limitations of uneven vaccination coverage [2,3,14]. Modelling and field evidence consistently indicate that achieving ≥ 70% immunity at the cluster level is essential for interrupting PPRV transmission [3,10,13,16]. Together, these findings emphasize the need for sustained high-coverage vaccination, strengthened surveillance, and rigorous post-vaccination evaluation to achieve and maintain PPR immunity for its eradication. The temporal distribution of PPR outbreaks reported across Indian states during 2023–2025 provides important insight into the epidemiological impact of the mass vaccination programme. A progressive decline in laboratory-confirmed sporadic outbreaks from 57 in 2023 to 20 in 2025 across the studied states reflects the cumulative protective effect of two consecutive annual vaccination rounds implemented under the National PPR-EP (Table 1). This reduction closely paralleled the observed increase in population immunity across states.

The most notable decline was observed in Telangana, where outbreaks decreased from 39 in 2023 to a single outbreak in 2025, corresponding with the highest recorded population immunity (>85%). Similarly, Andhra Pradesh reported no outbreaks in 2025 as population immunity approached 80%. States such as Odisha and Chhattisgarh, which maintained seroprevalence above 75%, also showed minimal sporadic outbreaks, reinforcing the importance of maintaining herd immunity above the critical threshold required for sustained prevention and control. In contrast, Karnataka and Maharashtra, despite seroprevalence near the herd immunity threshold (~72%), continued to report sporadic outbreaks during 2024–2025, suggesting that intensified surveillance activities, besides localized immunity gaps, at the epi-unit level may have permitted continued virus circulation, particularly in areas with high animal movement and trade/market location. Differences in vaccine rollout timing, uneven coverage among nomadic and semi-intensive production systems, and operational challenges likely contributed to transient immunity gaps that sustained localized outbreaks despite national vaccination efforts [3,25]. Similar operational challenges have also been documented in Ethiopia, including high herd mobility, rapid turnover of young stock, and lack of post-vaccination seromonitoring [26,27]. Molecular investigations confirmed exclusive circulation of PPRV lineage IV in recent outbreaks, with continued transmission facilitated by inter-regional movement and trade of infected animals [28]. Global experiences align with these observations. In Ethiopia, vaccinated herds with seroprevalence above 68% showed significantly fewer outbreaks than unvaccinated groups [29], while countries such as Morocco and Turkey achieved near-elimination of the disease following sustained high-coverage mass vaccination campaigns. These findings reinforce the importance of attaining and maintaining high population immunity for effective disease control and eradication.

The Indian experience also highlights the economic benefits of vaccination. In Karnataka, strategic vaccination substantially reduced outbreak burden and generated a benefit-to-cost ratio exceeding 18:1 through improved flock productivity and reduced economic losses [25]. Independent estimates further suggest that returns on investment in PPR vaccine research, development, and administration may exceed 40–50 fold under different outbreak scenarios [30]. These findings validate the economic viability of sustained annual vaccination campaigns. However, achieving uniform protection across all production systems, including nomadic and semi-intensive systems, remains essential. Operational limitations such as inadequate post-vaccination surveillance, uneven campaign implementation, lack of animal identification systems, and insufficient cross-border harmonization of vaccination activities continue to pose challenges for eradication efforts.

Globally, PPR affects more than 70 countries across Africa, the Middle East, and Asia, placing nearly 80% of the world’s small ruminant population at risk [31]. In India, the national PPR CP initiated in 2011 has substantially reduced outbreak incidence through mass vaccination and surveillance [2,25]. Nevertheless, sporadic outbreaks continue to occur in certain regions despite progressive control efforts, and recent sporadic outbreaks have confirmed continued circulation of lineage IV virus [28]. Spillover into wildlife, including the 2024 outbreak among captive deer in Assam [32], further emphasizes the need for sustained vaccination coverage and vigilant surveillance. Overall, the declining trend in outbreaks together with increasing population immunity provides strong evidence that strategic mass vaccination is effectively controlling PPR in India. However, continued efforts to close residual immunity gaps and maintain uniform herd immunity across all epi-units will be essential for achieving PPR-free status in India by 2030 in alignment with the GCES of PPR-GEP [4,10].

## 5. Interpretation of Findings and Limitations

The statistical analysis was descriptive and programme-oriented, as the study aimed to evaluate post-vaccination seroprevalence under the National PPR EP rather than identify individual-level risk factors. Seroprevalence was estimated at the population level, with comparisons across species, age, sex, and States/UTs to support programme monitoring. Although the sampling was multistage and hierarchical, mixed-effects modelling was not feasible because flock/household identifiers and key covariates (e.g., management practices and vaccination history) were not consistently available. Therefore, associations were assessed using chi-square (χ^2^) tests for exploratory comparisons. Potential clustering effects are acknowledged, and *p*-values interpreted with caution. The survey was conducted independently within each State/UT to achieve the target sample size; therefore, no national-level weighting was applied. Although sampling intensity was guided by state-wise small-ruminant density categories, uniform operational procedures were followed across all States/UTs. As the primary objective was programme monitoring rather than estimation of nationally weighted prevalence, findings are presented as programme-level estimates. Convenience-based selection of animals may have introduced selection bias, consistent with the operational sampling approach recommended under the GCES.

Further, seroprevalence estimated using c-ELISA provides an indirect measure of population exposure and should not be interpreted as direct protective immunity at the individual animal level. In this study, “population immunity” refers to the proportion of animals with detectable antibodies against PPR virus and serves as an operational indicator for monitoring vaccination programmes. Although c-ELISA positivity (>60 PI) correlates with VNT responses [17], it cannot distinguish vaccine-induced from infection-induced antibodies. In addition, apparent prevalence was reported without adjustment for test sensitivity and specificity, consistent with the objective of programme evaluation. Therefore, the serological findings were interpreted as indicators of vaccination coverage and population immune status rather than definitive measures of herd immunity.

The comparison between baseline (≈41%) and post-vaccination (≈72%) immunity or seroprevalence should be interpreted as a programme monitoring indicator rather than a formal estimate of vaccine effectiveness. Although both surveys were conducted by the same institute using identical WOAH/FAO GCES sampling protocols, laboratory methods, and analytical procedures, they represent independent nationwide cross-sectional surveys rather than longitudinal follow-up of the same animal populations. The baseline dataset, while serving as the official reference for implementation of the National PPR EP, has not yet been published independently. Consequently, the observed increase in seroprevalence provides evidence of improved population-level antibody prevalence following mass vaccination but should not be interpreted as direct evidence of individual-level vaccine-induced protection. Likewise, the observed reduction in reported outbreaks is considered consistent with vaccination activities rather than proof of causality. In the absence of control populations or longitudinal follow-up, causal inference regarding vaccine effectiveness is not possible, and the findings should be interpreted as programme-level epidemiological indicators.

## 6. Conclusions

The present study findings indicate that the desired animal level population immunity for eradication and herd protection can be achieved within two cycles of annual mass vaccination, although up to three to four cycles may be required in low-coverage or high-risk clusters. Population immunity against PPR varied considerably across states implementing the PPR EP, reflecting uneven historical vaccination coverage and operational challenges, with low seroprevalence immunity in some regions. These gaps emphasize the need for intensified mass vaccination coupled with active surveillance to establish and sustain PPR-free zones under the National Strategic Plan. Periodic serological surveillance should complement administrative vaccination coverage data to provide a more accurate assessment of population immunity, identify residual immunity gaps, and guide interventions necessary for successful PPR eradication. To accelerate eradication, mass vaccination of more than 95% of animals aged 3–4 months to increase vaccination coverage, with vaccine efficacy/vaccine effectiveness exceeding 85%, is essential to achieve desired 80% herd/population immunity. Post-vaccination evaluation surveys conducted 30–90 days after the initial and subsequent campaigns are critical for monitoring population immunity trends and guiding program adjustments.

Once adequate immunity is established and outbreaks become rare or sporadic, control efforts should transition to risk-based vaccination in high-risk areas such as border districts, animal markets, trade locations, and transit points, including the use of DIVA vaccine to differentiate infection in vaccinated animals. Zoning of PPR-risk regions, targeted vaccination, and continuous surveillance across epidemiological zones remain key components of eradication efforts. The results obtained in this study support evidence-based vaccination planning and regional prioritization to advance India’s goal of PPR freedom.

## Figures and Tables

**Table 1:** Details of the PPRV antibodies’ prevalence in small ruminants across 20 States/UTs of India after the Second Annual Mass Vaccination implemented under the National PPR Eradication Programme.

Name of States/Union Territories	No. of Districts in the State	No. of Taluks in the State	Vaccination Coverage (%) ^#^	No. of Villages/Epi-Units	No. of Serum Samples Screened	No. of Samples Positive in ELISA	Prevalence (%)	No. of Serum Samples Screened	No. of Samples Positive in ELISA	Prevalence (%)	Total No. of Serum Samples Screened	Total No. of Samples Positive in ELISA	Prevalence (%)	Officially Reported Passive PPR Laboratory Confirmed Outbreaks ^$^		
					Sheep			Goat			Total			2023	2024	2025
Andhra Pradesh	23	129	94.9	148	3064	2455	80.1	1871	1467	78.4	4935	3922	79.5	10	12	-
Assam	40	63	93	80	36	28	77.8	2021	1262	62.4	2057	1290	62.7	-	-	-
Bihar	33	94	99.6	107	84	53	63.1	3118	1816	58.2	3202	1869	58.4	-	1	-
Chandigarh *	1	1	100	4	6	5	83.3	98	89	90.8	104	94	90.4	-	-	-
Chhattisgarh	25	66	90.4	91	56	37	66.1	2644	1989	75.2	2700	2026	75	-	-	1
Goa *	2	16	≈90	54	16	12	75	1124	695	61.8	1140	707	62	-	1	1
Gujarat	25	62	99	75	449	351	78.2	1787	1347	75.4	2236	1698	75.9	3	1	-
Karnataka	22	74	90.9	129	3226	2574	79.8	1317	912	69.2	4543	3486	76.7	1	6	6
Ladakh	2	8	86	54	903	467	51.7	632	399	63.1	1535	866	56.4	-	-	-
Madhya Pradesh	35	63	96	74	25	14	56	2257	972	43.1	2282	986	43.2	2	2	2
Maharashtra	25	66	98.7	75	285	219	76.8	1637	1161	70.9	1922	1380	71.8	1	5	6
Meghalaya	7	23	≈85	43	71	39	54.9	974	552	56.7	1045	591	56.6	-	-	-
Mizoram *	9	21	75	54	-	-	-	1368	545	39.8	1368	545	39.8	1	-	-
Odisha	27	77	87.3	90	266	226	85	2384	1943	81.5	2650	2169	81.8	-	1	1
Puducherry	3	8	≈88	100	37	19	51.4	2651	1840	69.4	2688	1859	69.2	-	-	1
Punjab	18	34	100	66	210	142	67.6	1755	1298	74	1965	1440	73.3	-	1	-
Tamil Nadu	27	96	≈90	123	1162	930	80	3255	2748	84.4	4417	3678	83.3	-	2	1
Telangana	30	143	91	148	4150	3447	83.1	2976	2636	88.6	7126	6083	85.4	39	18	1
Uttarakhand	13	52	74	67	223	145	65	1751	1091	62.3	1974	1236	62.6	-	1	-
West Bengal	16	65	99.5	84	128	93	72.7	2116	1369	64.7	2244	1462	65.2	-	-	-
Grand Total	383	1161		1666	14,397	11,256	78.2	37,736	26,131	69.3	52,133	37,387	71.7	57	51	20
Chi-squared value (*χ*^2^): *p*-value and Prevalence at 95% CI					*χ*^2^: 548.8, *p* < 0.005		CI 95%: (77.6 to 78.9)	*χ*^2^: 2902.1, *p* < 0.005		CI 95%: (69.0 to 70.0)	*χ*^2^: 3758.9, *p* < 0.005		CI 95%: (71.0 to 72.4)			

* *χ*^2^ value was not calculated for Chandigarh, Goa and Mizoram while analysis association across sheep populations as their values were less than 5, and CI—confidence interval. ^#^ Indicates the proportion of the eligible sheep and goat population vaccinated under the programme, and ^$^ refers to outbreaks detected through the national passive surveillance system and subsequently confirmed by laboratory diagnosis. The passive surveillance system operates through routine field-level reporting of suspected PPR cases by State Animal Husbandry Departments, collection and submission of clinical samples from outbreak investigations, and laboratory confirmation through the designated national PPR diagnostic network. This system enables timely detection and confirmation of new outbreaks across states/UTs.

**Table 2:** Details of the PPRV antibody prevalence distribution (in different age groups of 6–12 Months, 1–2 Year and >2 Years) in small ruminants after the Second Annual Mass Vaccination implemented under the National PPR Eradication Programme.

Name of States/Union Territories	No. of Serum Samples Screened	No. of Samples Positive in ELISA	Prevalence (%)	No. of Serum Samples Screened	No. of Samples Positive in ELISA	Prevalence (%)	No. of Serum Samples Screened	No. of Samples Positive in ELISA	Prevalence (%)
	6–12 Months			1–2 Years			>2 Years		
Andhra Pradesh	1650	1287	78	1687	1332	79	1598	1303	81.5
Assam	683	433	63.4	693	445	64.2	681	412	60.5
Bihar	1066	639	59.9	1069	619	57.9	1067	611	57.3
Chandigarh	35	30	85.7	39	37	94.9	30	27	90
Chhattisgarh	927	687	74.1	904	683	75.6	869	656	75.5
Goa	403	233	57.8	383	245	64	354	229	64.7
Gujarat	744	546	73.4	765	593	77.5	727	559	76.9
Karnataka	1522	1138	74.8	1547	1184	76.5	1474	1164	79
Ladakh	514	278	54.1	475	270	56.8	546	318	58.2
Madhya Pradesh	759	345	45.5	754	332	44	769	309	40.2
Maharashtra	639	449	70.3	679	476	70.1	604	455	75.3
Meghalaya	366	225	61.5	367	212	57.8	312	154	49.4
Mizoram	547	229	41.9	403	151	37.5	418	165	39.5
Odisha	857	696	81.2	896	731	81.6	897	742	82.7
Puducherry	1320	955	72.3	697	448	64.3	671	456	68
Punjab	659	454	68.9	666	489	73.4	640	497	77.7
Tamil Nadu	1487	1198	80.6	1476	1252	84.8	1454	1228	84.5
Telangana	2439	2057	84.3	2380	2028	85.2	2307	1998	86.6
Uttarakhand	659	405	61.5	677	430	63.5	638	401	62.9
West Bengal	717	455	63.5	750	474	63.2	777	533	68.6
Grand Total	18,002	12,748	70.8	17,316	12,440	71.8	16,842	12,226	72.6
Chi-squared value (*χ*^2^): *p*-value and Prevalence at 95% CI	*χ*^2^: 1084.3, *p* < 0.005		CI 95%: 70.0 to 72.0	*χ*^2^: 1263.5, *p* < 0.005		CI 95%: 72.0 to 73.0	*χ*^2^: 1505.0, *p* < 0.005		CI 95%: 72.0 to 73.0

CI—confidence interval.

## Data Availability

The data presented in this study are available in the manuscript and in the Supplementary Files. All datasets supporting our findings are available at the WOAH Reference Laboratory for PPR, ICAR-NIVEDI, Bengaluru, on reasonable request from the corresponding author.

## Supplementary Materials

The following supporting information can be downloaded at: https://www.mdpi.com/article/10.3390/v18080839/s1, Table S1: Sheep and Goat Population (in Millions) Across Selected Indian States Based on the 20th Livestock Census (2019). Tables S2–S21: Details of the population immunity status PPR (% Seroprevalence) in small ruminants in various districts of studied states in India. Table S22: Details of the PPRV antibodies prevalence distribution (in ≤30%, 31–70% and >70% prevalence) in small ruminants. Table S23: Chi-squared results of PPRV antibody prevalence in small ruminants.
