# Supplementary material for: Assessment of Population Immunity Against Peste des Petits Ruminants in Goats and Sheep in India After the Second Annual Mass Vaccination Implemented Under the National PPR Eradication Programme
Source: Viruses. 2026 Jul 30;18(8):839. doi: 10.3390/v18080839 (PMC13517470; doi:10.3390/v18080839)
Supplement: Supplementary file 1 [file viruses-18-00839-s001.zip › viruses-4372714-supplementary.pdf]

Supplementary Documents

Title: Assessment of Population Immunity against Peste des Petits Ruminants in Goats and Sheep in India after the Second Annual Mass Vaccination implemented under the National PPR Eradication Programme

Table S1: Sheep and Goat Population (in Millions) Across Selected Indian States Based on the 20th Livestock Census (2019)

State	Sheep	Goat
Andhra Pradesh	17.63	5.52
Assam	0.33	4.32
Bihar	0.21	12.82
Chandigarh	0	0.001
Chhattisgarh	0.18	4.01
Goa	0	0.01
Gujarat	1.79	4.87
Karnataka	11.05	6.17
Ladakh	0.04	0.03
Madhya Pradesh	0.85	11.06
Maharashtra	2.68	10.6
Meghalaya	0.03	0.45
Mizoram	0	0.04
Odisha	1.28	6.39
Puducherry	0	0.07
Punjab	0.09	0.35
Tamil Nadu	4.5	9.89
Telangana	19.06	4.93
Uttarakhand	0.28	1.37
West Bengal	0.95	16.28
**Grand Total**	**60.95**	**99.18**

**Sheep population in Goa (8 animals), Puducherry (2,445 animals), Chandigarh (approximately 20 animals), and Mizoram (approximately 3,000 animals) are less than 0.01 million and therefore appear as approximately zero when rounded.*

Table S2. Details of the population immunity status PPR (% Seroprevalence) in small ruminants in the state of Andhra Pradesh

Name of the Districts in Andhra Pradesh	No. of Taluks in the State	No. of Village/ Epi-Units	Sheep: No. of serum samples screened	Sheep: No. of samples positive in ELISA	Sheep: Prevalence (%)	Goat: No. of serum samples screened	Goat: No. of samples positive in ELISA	Goat: Prevalence (%)	Total: No. of serum samples screened	Total: No. of samples positive in ELISA	Total: Prevalence (%)
Anantapur	9	9	216	144	66.7	78	45	57.7	294	189	64.3
Chittoor	3	5	114	109	95.6	51	39	76.5	165	148	89.7
East Godavari	5	5	42	41	97.6	119	105	88.2	161	146	90.7
Guntur	1	1	25	-	-	8	-	-	33	-	-
Kurnool	7	10	290	237	81.7	40	30	75	330	267	80.9
Prakasam	8	9	273	200	73.3	14	8	57.1	287	208	72.5
SPSR Nellore	10	14	191	144	75.4	259	179	69.1	450	323	71.8
Srikakulam	7	9	158	112	70.9	138	116	84.1	296	228	77
Visakhapatanam	2	2	39	32	82.1	27	27	100	66	59	89.4
Vizianagaram	7	10	131	114	87	191	154	80.6	322	268	83.2
Tirupati	17	21	420	301	71.7	268	202	75.4	688	503	73.1
Kakinada	1	1	8	7	87.5	25	23	92	33	30	90.9
Palnadu	6	6	118	117	99.2	80	78	97.5	198	195	98.5
Bapatla	2	2	33	14	42.4	33	33	100	66	47	71.2
NTR	3	5	125	117	93.6	40	35	87.5	165	152	92.1
Eluru	5	5	102	85	83.3	62	42	67.7	164	127	77.4
Nandyal	8	8	225	199	88.4	39	26	66.7	264	225	85.2
Alluri Sithrama Raju at Pderu	5	5	27	26	96.3	138	118	85.5	165	144	87.3
Putparthy	3	3	95	85	89.5	2	2	100	97	87	89.7
Parvathipuram	6	6	67	55	82.1	131	95	72.5	198	150	75.8
Krishna	2	2	55	40	72.7	10	7	70	65	47	72.3
Annamaya	8	8	255	222	87.1	9	9	100	264	231	87.5
Anakakpalli	5	5	55	54	98.2	109	94	86.2	164	148	90.2
Grand Total, Prevalence at 95% CI	130	151	3064	2455	80.1 CI 95%: (79 to 82)	1871	1467	78.4 CI 95%: (77 to 80)	4935	3922	79.5 CI 95%: (78 to 81)

Table S3. Details of the population immunity status PPR (% Seroprevalence) in small ruminants in the state of Assam

Name of the Districts in Assam	No. of Taluks in the State	No. of Village/ Epi-Units	No. of serum samples screened	No. of samples positive in ELISA	Prevalence (%)	No. of serum samples screened	No. of samples positive in ELISA	Prevalence (%)	No. of serum samples screened	No. of samples positive in ELISA	Prevalence (%)
			Sheep			Goat			Total		
Baksa	3	4	-	-	-	94	71	75.5	94	71	75.5
Bongaigaon	3	4	-	-	-	101	70	69.3	101	70	69.3
Cachar	3	4	12	10	83.3	90	71	78.9	102	81	79.4
Chirang	1	1	-	-	-	24	21	87.5	24	21	87.5
Darrang	1	1	-	-	-	27	24	88.9	27	24	88.9
Dhemaji	2	2	-	-	-	45	11	24.4	45	11	24.4
Goalpara	2	3	9	3	33.3	62	33	53.2	71	36	50.7
Golaghat	1	1	-	-	-	26	24	92.3	26	24	92.3
Hailakandi	1	1	-	-	-	27	0	0	27	0	0
Karbi Anglong	2	6	-	-	-	151	68	45	151	68	45
Karimganj	2	2	3	3	100	45	41	91.1	48	44	91.7
Kokrajhar	3	4	-	-	-	108	77	71.3	108	77	71.3
Lakhimpur	3	3	-	-	-	78	68	87.2	78	68	87.2
Majuli	1	1	-	-	-	25	22	88	25	22	88
Morigaon	1	1	-	-	-	25	6	24	25	6	24
Nagaon	2	3	-	-	-	81	45	55.6	81	45	55.6
Udalguri	1	1	-	-	-	27	26	96.3	27	26	96.3
Sonitpur	3	3	-	-	-	81	58	71.6	81	58	71.6
Udalguri	2	2	-	-	-	54	37	68.5	54	37	68.5
Sonitpur	1	1	-	-	-	27	26	96.3	27	26	96.3
Nagaon	2	2	-	-	-	54	14	25.9	54	14	25.9
Udalguri	1	1	-	-	-	27	24	88.9	27	24	88.9
Jorhat	2	2	-	-	-	54	34	63	54	34	63
Dibrugarh	3	4	-	-	-	100	55	55	100	55	55
Biswanath	2	2	-	-	-	51	27	52.9	51	27	52.9
Barpeta	1	1	-	-	-	27	11	40.7	27	11	40.7
Kamrup	2	2	-	-	-	54	37	68.5	54	37	68.5
Hojai	1	1	-	-	-	27	3	11.1	27	3	11.1
Jorhat	1	1	-	-	-	27	25	92.6	27	25	92.6
Nalbari	1	1	-	-	-	27	18	66.7	27	18	66.7
Nalbari	2	3	-	-	-	81	38	46.9	81	38	46.9
Dhemaji	1	1	-	-	-	24	5	20.8	24	5	20.8
Dibrugarh	2	2	-	-	-	51	39	76.5	51	39	76.5
Dhubri	1	1	12	12	100	15	6	40	27	18	66.7
Tinsukia	1	1	-	-	-	24	0	0	24	0	0

Sibsagar	1	1	-	-	-	21	18	85.7	21	18	85.7
Soraideu	1	1	-	-	-	24	24	100	24	24	100
West Karbi Anglong	1	2	-	-	-	54	30	55.6	54	30	55.6
Biswanath	1	1	-	-	-	27	26	96.3	27	26	96.3
Morigaon	2	2	-	-	-	54	29	53.7	54	29	53.7
Grand Total, Prevalence at 95% CI	67	80	36	28	77.8 CI 95%: (64 to 91)	2021	1262	62.4 CI 95%: (60 to 65)	2057	1290	62.7 CI 95%: (61 to 65)

Table S4. Details of the population immunity status PPR (% Seroprevalence) in small ruminants in state of Bihar

Name of the Districts in Bihar	No. of Taluks in the State	No. of Village/ Epi-Units	No. of serum samples screened	No. of samples positive in ELISA	Prevalence (%)	No. of serum samples screened	No. of samples positive in ELISA	Prevalence (%)	No. of serum samples screened	No. of samples positive in ELISA	Prevalence (%)
			Sheep			Goat			Total		
Araria	4	4	-	-	-	120	53	44.2	120	53	44.2
Arwal	1	2	-	-	-	60	41	68.3	60	41	68.3
Aurangabad	3	3	-	-	-	60	59	98.3	60	59	98.3
Banka	4	4	-	-	-	120	80	66.7	120	80	66.7
Buxar	1	1	15	0	0	15	0	0	30	0	0
Darbhanga	3	3	-	-	-	90	89	98.9	90	89	98.9
Gaya	5	8	3	3	100	237	219	92.4	240	222	92.5
Gopalganj	1	1	-	-	-	30	19	63.3	30	19	63.3
Jamui	5	6	-	-	-	179	172	96.1	179	172	96.1
Kaimur (Bhabua)	1	1	-	-	-	30	8	26.7	30	8	26.7
Katihar	2	3	-	-	-	90	60	66.7	90	60	66.7
Khagaria	1	1	-	-	-	30	0	0	30	0	0
Kishanganj	1	1	-	-	-	30	1	3.3	30	1	3.3
Lakhisarai	1	1	-	-	-	30	15	50	30	15	50
Madhepura	1	1	-	-	-	30	14	46.7	30	14	46.7
Madhubani	5	5	-	-	-	150	79	52.7	150	79	52.7
Munger	3	3	-	-	-	90	54	60	90	54	60
Muzaffarpur	8	8	-	-	-	239	93	38.9	239	93	38.9
Nalanda	2	2	30	28	93.3	30	25	83.3	60	53	88.3
Nawada	4	5	-	-	-	148	117	79.1	148	117	79.1
Pashchim Champaran	4	4	-	-	-	120	26	21.7	120	26	21.7
Purbi Champaran	9	12	-	-	-	359	232	64.6	359	232	64.6
Purnia	2	2	-	-	-	60	54	90	60	54	90
Rohtas	2	2	9	0	0	51	0	0	60	0	0
Saharsa	2	2	-	-	-	60	30	50	60	30	50
Samastipur	4	4	-	-	-	120	50	41.7	120	50	41.7
Sheikhpura	2	3	24	22	91.7	65	38	58.5	89	60	67.4
Sitamarhi	4	4	-	-	-	120	95	79.2	120	95	79.2
Supaul	1	1	-	-	-	28	19	67.9	28	19	67.9
Vaishali	6	7	3	0	0	207	19	9.2	210	19	9
Siwan	2	2	-	-	-	60	0	0	60	0	0
Bhagapur	1	1	-	-	-	30	25	83.3	30	25	83.3
Saran	1	1	-	-	-	30	30	100	30	30	100
Grand Total, Prevalence at 95% CI	96	108	84	53	63.1 CI 95%: (53 to 73)	3118	1816	58.2 CI 95%: (57 to 60)	3202	1869	58.4 CI 95%: (57 to 60)

Table S5. Details of the population immunity status PPR (% Seroprevalence) in small ruminants in the state of Chandigarh

Name of the Districts in Chandigarh	No. of Taluks in the State	No. of Village/ Epi-Units	No. of serum samples screened	No. of samples positive in ELISA	Prevalence (%)	No. of serum samples screened	No. of samples positive in ELISA	Prevalence (%)	No. of serum samples screened	No. of samples positive in ELISA	Prevalence (%)
			Sheep			Goat			Total		
Chandigarh	1	4	6	5	83.3	98	89	90.8	104	94	90.4
Grand Total, Prevalence at 95% CI	1	4	6	5	83.3 CI 95%: (54 to 113)	98	89	90.8 CI 95%: (85 to 97)	104	94	90.4 CI 95%: (85 to 96)

Table S6. Details of the population immunity status PPR (% Seroprevalence) in small ruminants in the state of Chhattisgarh

Name of the Districts in Chhattisgarh	No. of Taluks in the State	No. of Village/ Epi-Units	Sheep: No. of serum samples screened	Sheep: No. of samples positive in ELISA	Sheep: Prevalence (%)	Goat: No. of serum samples screened	Goat: No. of samples positive in ELISA	Goat: Prevalence (%)	Total: No. of serum samples screened	Total: No. of samples positive in ELISA	Total: Prevalence (%)
Balrampur	5	7	-	-	-	210	126	60	210	126	60
Bastar	2	2	-	-	-	60	57	95	60	57	95
Bijapur	6	10	-	-	-	30	21	70	30	21	70
Bilaspur	1	1	-	-	-	30	19	63.3	30	19	63.3
Dantewada	1	1	-	-	-	30	29	96.7	30	29	96.7
Dhamtari	2	3	-	-	-	90	51	56.7	90	51	56.7
Durg	1	1	-	-	-	30	22	73.3	30	22	73.3
Gariyaband	2	3	26	16	61.5	64	45	70.3	90	61	67.8
Janjgir-Champa	1	1	-	-	-	30	15	50	30	15	50
Jashpur	7	13	-	-	-	388	282	72.7	388	282	72.7
Kanker	6	7	-	-	-	210	197	93.8	210	197	93.8
Kondagaon	3	4	30	21	70	90	79	87.8	120	100	83.3
Korba	3	5	-	-	-	149	105	70.5	149	105	70.5
Korea	5	5	-	-	-	150	96	64	150	96	64
Mahasamund	2	3	-	-	-	90	81	90	90	81	90
Narayanpur	1	2	-	-	-	60	49	81.7	60	49	81.7
Raigarh	3	3	-	-	-	90	81	90	90	81	90
Raipur	1	1	-	-	-	30	22	73.3	30	22	73.3
Rajnandgaon	3	4	-	-	-	120	112	93.3	120	112	93.3
Sukma	1	4	-	-	-	97	81	83.5	97	81	83.5
Surajpur	3	5	-	-	-	150	124	82.7	150	124	82.7
Surguja	6	7	-	-	-	208	117	56.3	208	117	56.3
Gorela-pendra-marwahi	2	2	-	-	-	59	53	89.8	59	53	89.8
Bametara	1	1	-	-	-	29	0	0	29	0	0
Balodabazar-Bhatapara	3	5	-	-	-	150	125	83.3	150	125	83.3
Grand Total, Prevalence at 95% CI	71	100	56	37	66.1 CI 95%: (54 to 78)	2644	1989	75.2 CI 95%: (74 to 77)	2700	2026	75 CI 95%: (73 to 77)

Table S7. Details of the population immunity status PPR (% Seroprevalence) in small ruminants in the state of Goa

Name of the Districts in Goa	No. of Taluks in the State	No. of Village/ Epi-Units	No. of serum samples screened	No. of samples positive in ELISA	Prevalence (%)	No. of serum samples screened	No. of samples positive in ELISA	Prevalence (%)	No. of serum samples screened	No. of samples positive in ELISA	Prevalence (%)
				Sheep			Goat			Total	
South Goa	9	27	-	-	-	537	349	65	537	349	65
North Goa	7	26	16	12	75	587	346	58.9	603	358	59.4
Grand Total	16	53	16	12	75 CI 95%: (54 to 96)	1124	695	61.8 CI 95%: (59 to 65)	1140	707	62 CI 95%: (59 to 65)

Table S8. Details of the population immunity status PPR (% Seroprevalence) in small ruminants in the state of Gujarat

Name of the Districts in Gujarat	No. of Taluks in the State	No. of Village/ Epi-Units	Sheep: No. of serum samples screened	Sheep: No. of samples positive in ELISA	Sheep: Prevalence (%)	Goat: No. of serum samples screened	Goat: No. of samples positive in ELISA	Goat: Prevalence (%)	Total: No. of serum samples screened	Total: No. of samples positive in ELISA	Total: Prevalence (%)
Anand	2	2	12	12	100	48	47	97.9	60	59	98.3
Arvalli	2	2	18	13	72.2	39	35	89.7	57	48	84.2
Bharuch	1	2	-	-	-	60	46	76.7	60	46	76.7
Bhavnagar	3	4	91	75	82.4	21	21	100	112	96	85.7
Botad	1	1	24	24	100	6	6	100	30	30	100
Devbhumi Dwarka	1	1	24	18	75	6	1	16.7	30	19	63.3
Dohad	5	5	-	-	-	150	102	68	150	102	68
Gandhinagar	1	1	-	-	-	30	30	100	30	30	100
Jamnagar	3	3	36	28	77.8	54	35	64.8	90	63	70
Junagadh	1	1	18	17	94.4	12	9	75	30	26	86.7
Kachchh	3	4	54	44	81.5	69	56	81.2	123	100	81.3
Kheda	2	4	7	7	100	113	90	79.6	120	97	80.8
Mahisagar	1	1	-	-	-	30	6	20	30	6	20
Morbi	3	3	27	17	63	63	54	85.7	90	71	78.9
Narmada	2	2	-	-	-	58	48	82.8	58	48	82.8
Rajkot	2	2	45	38	84.4	15	12	80	60	50	83.3
Vadodara	3	4	-	-	-	120	102	85	120	102	85
Valsad	2	2	-	-	-	60	32	53.3	60	32	53.3
Patan	1	1	-	-	-	30	1	3.3	30	1	3.3
SabarKantha	2	2	6	1	16.7	53	23	43.4	59	24	40.7
BanasKantha	9	10	86	57	66.3	213	176	82.6	299	233	77.9
Mehsana	5	7	1	0	0	209	175	83.7	210	175	83.3
PanchMahals	5	8	-	-	-	238	170	71.4	238	170	71.4
Dang	1	1	-	-	-	30	24	80	30	24	80
Chhota Udepur	1	2	-	-	-	60	46	76.7	60	46	76.7
Grand Total, Prevalence at 95% CI	62	75	449	351	78.2 CI 95%: (74 to 82)	1787	1347	75.4 CI 95%: (73 to 77)	2236	1698	75.9 CI 95%: (74 to 78)

Table S9. Details of the population immunity status PPR (% Seroprevalence) in small ruminants in the state of Karnataka

Name of the Districts in Karnataka	No. of Taluks in the State	No. of Village/ Epi-Units	Sheep			Goat			Total		
			No. of serum samples screened	No. of samples positive in ELISA	Prevalence (%)	No. of serum samples screened	No. of samples positive in ELISA	Prevalence (%)	No. of serum samples screened	No. of samples positive in ELISA	Prevalence (%)
Bidar	4	6	66	46	69.7	117	104	88.9	183	150	82
Bijapur	6	10	180	101	56.1	143	58	40.6	323	159	49.2
Dharwad	1	1	-	-	-	35	23	65.7	35	23	65.7
Gadag	3	4	93	53	57	48	16	33.3	141	69	48.9
Gulbarga	7	13	217	186	85.7	243	198	81.5	460	384	83.5
Hassan	3	7	211	185	87.7	37	37	100	248	222	89.5
Haveri	4	7	126	85	67.5	124	56	45.2	250	141	56.4
Kolar	5	10	352	271	77	5	5	100	357	276	77.3
Koppal	3	7	251	233	92.8	-	-	-	251	233	92.8
Mandya	4	7	175	145	82.9	74	35	47.3	249	180	72.3
Mysore	4	4	70	51	72.9	74	66	89.2	144	117	81.3
Ramanagara	3	5	89	59	66.3	88	53	60.2	177	112	63.3
Yadgir	2	3	90	65	72.2	18	18	100	108	83	76.9
Davanagere	3	6	161	123	76.4	55	39	70.9	216	162	75
Raichur	5	7	196	145	74	53	45	84.9	249	190	76.3
Bagalkote	5	7	218	148	67.9	28	19	67.9	246	167	67.9
Shivamogga	1	1	6	6	100	27	27	100	33	33	100
Chikkaballapura	6	17	576	546	94.8	33	30	90.9	609	576	94.6
Chamarajanagara	2	4	76	70	92.1	66	51	77.3	142	121	85.2
Chickmagalur	2	2	50	41	82	21	15	71.4	71	56	78.9
Bangalore Urban	1	1	19	11	57.9	17	6	35.3	36	17	47.2
Vijayanagar	1	1	4	4	100	11	11	100	15	15	100
Grand Total, Prevalence at 95% CI	75	130	3226	2574	79.8 CI 95%: (78 to 81)	1317	912	69.2 CI 95%: (67 to 72)	4543	3486	76.7 CI 95%: (76 to 78)

Table S10. Details of the population immunity status PPR (% Seroprevalence) in small ruminants in the state of Ladakh

Name of the Districts in Ladakh	No. of Taluks in the State	No. of Village/ Epi-Units	Sheep			Goat			Total		
			No. of serum samples screened	No. of samples positive in ELISA	Prevalence (%)	No. of serum samples screened	No. of samples positive in ELISA	Prevalence (%)	No. of serum samples screened	No. of samples positive in ELISA	Prevalence (%)
Kargil	4	37	666	333	50	377	217	57.6	1043	550	52.7
Leh Ladakh	4	17	237	134	56.5	255	182	71.4	492	316	64.2
Grand Total, Prevalence at 95% CI	8	54	903	467	51.7CI 95%: (48 to 55)	632	399	63.1 CI 95%: (59 to 67)	1535	866	56.4 CI 95%: (54 to 59)

Table S11. Details of the population immunity status PPR (% Seroprevalence) in small ruminants in the state of Madhya Pradesh

Name of the Districts in Madhya Pradesh	No. of Taluks in the State	No. of Village/ Epi-Units	Sheep			Goat			Total		
			No. of serum samples screened	No. of samples positive in ELISA	Prevalence (%)	No. of serum samples screened	No. of samples positive in ELISA	Prevalence (%)	No. of serum samples screened	No. of samples positive in ELISA	Prevalence (%)
Agar Malwa	1	1				30	25	83.3	30	25	83.3
Alirajpur	3	3	-	-	-	90	32	35.6	90	32	35.6
Barwani	2	2	-	-	-	60	20	33.3	60	20	33.3
Betul	2	2	-	-	-	60	11	18.3	60	11	18.3
Bhind	1	1	-	-	-	30	0	0	30	0	0
Chhatarpur	4	5	-	-	-	150	87	58	150	87	58
Chhindwara	7	7	-	-	-	183	146	79.8	183	146	79.8
Damoh	1	1	-	-	-	30	12	40	30	12	40
Datia	1	1	-	-	-	29	29	100	29	29	100
Dewas	2	2	-	-	-	60	17	28.3	60	17	28.3
Dhar	3	4	-	-	-	120	54	45	120	54	45
Gwalior	2	4	10	8	80	110	97	88.2	120	105	87.5
Jhabua	2	3	-	-	-	90	8	8.9	90	8	8.9
Katni	1	1	-	-	-	30	8	26.7	30	8	26.7
Khargone	2	3	-	-	-	90	37	41.1	90	37	41.1
Mandsaur	3	3	-	-	-	90	78	86.7	90	78	86.7
Narsinghpur	1	1	-	-	-	30	6	20	30	6	20
Rajgarh	1	1	-	-	-	30	30	100	30	30	100
Ratlam	1	2	-	-	-	60	16	26.7	60	16	26.7
Rewa	1	1	-	-	-	30	8	26.7	30	8	26.7
Satna	2	2	-	-	-	60	41	68.3	60	41	68.3
Sehore	1	1	-	-	-	30	0	0	30	0	0
Shahdol	1	2	-	-	-	60	12	20	60	12	20
Sheopur	1	1	-	-	-	30	5	16.7	30	5	16.7
Shivpuri	3	4	6	0	0	114	16	14	120	16	13.3
Sidhi	1	1	-	-	-	30	0	0	30	0	0
Ujjain	3	4	-	-	-	120	62	51.7	120	62	51.7
Umaria	1	1	-	-	-	60	1	1.7	60	1	1.7

Singrauli	1	2	-	-	-	60	9	15	60	9	15
Mandla	1	1	-	-	-	30	3	10	30	3	10
Dindori	1	1	-	-	-	30	20	66.7	30	20	66.7
Tikamgarh	3	3	-	-	-	90	2	2.2	90	2	2.2
Dindori	1	1	-	-	-	60	27	45	60	27	45
Seoni	1	1	-	-	-	60	50	83.3	60	50	83.3
Butaria (NP)	1	1	9	6	66.7	21	3	14.3	30	9	30
Grand Total, Prevalence at 95% CI	63	74	25	14	56 CI 95%: (37 to 75)	2257	972	43.1 CI 95%: (41 to 45)	2282	986	43.2 CI 95%: (41 to 45)

Table S12. Details of the population immunity status PPR (% Seroprevalence) in small ruminants in the state of Maharashtra

Name of the Districts in Maharashtra	No. of Taluks in the State	No. of Village/ Epi-Units	Sheep			Goat			Total		
			No. of serum samples screened	No. of samples positive in ELISA	Prevalence (%)	No. of serum samples screened	No. of samples positive in ELISA	Prevalence (%)	No. of serum samples screened	No. of samples positive in ELISA	Prevalence (%)
Ahmednagar	7	7	27	24	88.9	152	83	54.6	179	107	59.8
Amravati	4	4	40	38	95	65	65	100	105	103	98.1
Bhandara	1	1	15	4	26.7	12	4	33.3	27	8	29.6
Chandrapur	1	1	11	9	81.8	16	12	75	27	21	77.8
Gadchiroli	3	4	-	-	-	87	82	94.3	87	82	94.3
Gondia	2	2	-	-	-	54	4	7.4	54	4	7.4
Jalgaon	1	1	-	-	-	20	18	90	20	18	90
Nagpur	2	2	8	7	87.5	21	12	57.1	29	19	65.5
Nandurbar	2	2	-	-	-	54	36	66.7	54	36	66.7
Nashik	5	5	-	-	-	129	82	63.6	129	82	63.6
Pune	2	3	21	9	42.9	57	47	82.5	78	56	71.8
Satara	4	4	-	-	-	128	89	69.5	128	89	69.5
Solapur	5	8	70	58	82.9	146	117	80.1	216	175	81
Wardha	2	2	-	-	-	51	48	94.1	51	48	94.1
Palghar	2	4	-	-	-	96	43	44.8	96	43	44.8
Latur	1	1	-	-	-	27	18	66.7	27	18	66.7
Osmanabad	1	1	-	-	-	27	20	74.1	27	20	74.1
Yavatmal	5	5	76	66	86.8	70	56	80	146	122	83.6
Hingoli	3	3	-	-	-	76	62	81.6	76	62	81.6
Washim	2	2	-	-	-	54	30	55.6	54	30	55.6
Aurangabad	3	3	-	-	-	27	21	77.8	27	21	77.8
Beed	4	5	17	4	23.5	86	36	41.9	103	40	38.8
Nanded	3	3	-	-	-	81	80	98.8	81	80	98.8
Jalna	3	3	-	-	-	81	78	96.3	81	78	96.3
Kolhapur	1	1	-	-	-	20	18	90	20	18	90
Grand Total, Prevalence at 95% CI	69	77	285	219	76.8 CI 95%: (72 to 82)	1637	1161	70.9 CI 95%: (69 to 73)	1922	1380	71.8 CI 95%: (70 to 74)

Table S13. Details of the population immunity status PPR (% Seroprevalence) in small ruminants in the state of Meghalaya

Name of the Districts in Meghalaya	No. of Taluks in the State	No. of Village / Epi-Units	Sheep: No. of serum samples screened	Sheep: No. of samples positive in ELISA	Sheep: Prevalence (%)	Goat: No. of serum samples screened	Goat: No. of samples positive in ELISA	Goat: Prevalence (%)	Total: No. of serum samples screened	Total: No. of samples positive in ELISA	Total: Prevalence (%)
West Khasi Hills	4	7	20	19	95	154	142	92.2	174	161	92.5
West Jaintia Hills	2	3	9	3	33.3	68	23	33.8	77	26	33.8
South West Garo Hills	4	15	15	10	66.7	357	164	45.9	372	174	46.8
East Garo Hills	4	4	5	3	60	98	80	81.6	103	83	80.6
West Garo Hills	5	8	22	4	18.2	160	98	61.3	182	102	56
South Garo Hills	3	5	-	-	-	114	22	19.3	114	22	19.3
East Khasi Hills	1	1	-	-	-	23	23	100	23	23	100
Grand Total, Prevalence at 95% CI	23	43	71	39	54.9 CI 95%: (43 to 67)	974	552	56.7 CI 95%: (54 to 60)	1045	591	56.6 CI 95%: (54 to 60)

Table S14. Details of the population immunity status PPR (% Seroprevalence) in small ruminants in the state of Mizoram

Name of the Districts in Mizoram	No. of Taluks in the State	No. of Village/ Epi-Units	Sheep: No. of serum samples screened	Sheep: No. of samples positive in ELISA	Sheep: Prevalence (%)	Goat: No. of serum samples screened	Goat: No. of samples positive in ELISA	Goat: Prevalence (%)	Total: No. of serum samples screened	Total: No. of samples positive in ELISA	Total: Prevalence (%)
Lawngtlai	1	2	51	25	49	-	-	-	51	25	49
Mamit	3	4	93	62	66.7	-	-	-	93	62	66.7
Serchhip	2	3	86	51	59.3	-	-	-	86	51	59.3
Kolasib	6	10	250	61	24.4	-	-	-	250	61	24.4
Lunglei	3	11	272	72	26.5	-	-	-	272	72	26.5
Siaha	2	11	297	181	60.9	-	-	-	297	181	60.9
Aizawl	2	6	128	26	20.3	-	-	-	128	26	20.3
Lawngtliai	1	2	48	1	2.1	-	-	-	48	1	2.1
Champhai	2	5	143	66	46.2	-	-	-	143	66	46.2
Grand Total, Prevalence at 95% CI	22	54	1368	545	39.8 CI 95%: (37 to 42)	0	0		1368	545	39.8 CI 95%: (37 to 42)

Table S15. Details of the population immunity status PPR (% Seroprevalence) in small ruminants in the state of Odisha

Name of the Districts in Odisha	No. of Taluks in the State	No. of Village/ Epi-Units	Sheep			Goat			Total		
			No. of serum samples screened	No. of samples positive in ELISA	Prevalence (%)	No. of serum samples screened	No. of samples positive in ELISA	Prevalence (%)	No. of serum samples screened	No. of samples positive in ELISA	Prevalence (%)
Anugul	4	7	16	14	87.5	184	158	85.9	200	172	86
Bargarh	2	2	-	-	-	60	41	68.3	60	41	68.3
Boudh	2	2	26	24	92.3	27	24	88.9	53	48	90.6
Deogarh	1	1	-	-	-	26	26	100	26	26	100
Dhenkanal	3	3	15	11	73.3	75	56	74.7	90	67	74.4
Gajapati	1	1	30	30	100				30	30	100
Ganjam	4	4	54	47	87	66	61	92.4	120	108	90
Jharsuguda	1	1	-	-	-	30	22	73.3	30	22	73.3
Kalahandi	2	2	12	9	75	48	42	87.5	60	51	85
Kandhamal	5	6	-	-	-	180	166	92.2	180	166	92.2
Kendrapara	1	1	-	-	-	30	27	90	30	27	90
Khordha	2	2	-	-	-	60	56	93.3	60	56	93.3
Koraput	2	2	32	25	78.1	26	18	69.2	58	43	74.1
Malkangiri	1	1	-	-	-	30	23	76.7	30	23	76.7
Mayurbhanj	9	15	-	-	-	439	359	81.8	439	359	81.8
Nabarangpur	4	4	55	40	72.7	62	44	71	117	84	71.8
Nayagarh	1	1	-	-	-	27	26	96.3	27	26	96.3
Puri	2	2	-	-	-	60	51	85	60	51	85
Rayagada	2	2	-	-	-	60	59	98.3	60	59	98.3
Sambalpur	2	2	-	-	-	60	56	93.3	60	56	93.3
Sonepur	3	3	20	20	100	69	41	59.4	89	61	68.5
Sundargarh	6	6	-	-	-	180	132	73.3	180	132	73.3
Bolangir	4	4	-	-	-	120	117	97.5	120	117	97.5
Jagatsinghpur	1	1	-	-	-	30	11	36.7	30	11	36.7
Jajpur	2	2	-	-	-	60	47	78.3	60	47	78.3
Keonjhar	6	8	-	-	-	238	160	67.2	238	160	67.2
Balasore	4	5	6	6	100	137	120	87.6	143	126	88.1
Grand Total, Prevalence at 95% CI	77	90	266	226	85 CI 95%: (81 to 89)	2384	1943	81.5 CI 95%: (80 to 83)	2650	2169	81.8 CI 95%: (80 to 83)

Table S16. Details of the population immunity status PPR (% Seroprevalence) in small ruminants in the state of Puducherry

Name of the Districts in Puducherry	No. of Taluks in the State	No. of Village/ Epi-Units	No. of serum samples screened	No. of samples positive in ELISA	Prevalence (%)	No. of serum samples screened	No. of samples positive in ELISA	Prevalence (%)	No. of serum samples screened	No. of samples positive in ELISA	Prevalence (%)
			Sheep			Goat			Total		
Karaikal	2	39	-	-	-	1052	717	68.2	1052	717	68.2
Pondicherry	6	59	37	19	51.4	1545	1084	70.2	1582	1103	69.7
Yanam	1	2	-	-	-	54	39	72.2	54	39	72.2
Grand Total, Prevalence at 95% CI	9	100	37	19	51.4 CI 95%: (35 to 67)	2651	1840	69.4 CI 95%: (68 to 71)	2688	1859	69.2 CI 95%: (67 to 71)

Table S17. Details of the population immunity status PPR (% Seroprevalence) in small ruminants in the state of Punjab

Name of the Districts in Punjab	No. of Taluks in the State	No. of Village/ Epi-Units	Sheep: No. of serum samples screened	Sheep: No. of samples positive in ELISA	Sheep: Prevalence (%)	Goat: No. of serum samples screened	Goat: No. of samples positive in ELISA	Goat: Prevalence (%)	Total: No. of serum samples screened	Total: No. of samples positive in ELISA	Total: Prevalence (%)
Barnala	2	2	-	-	-	60	7	11.7	60	7	11.7
Bathinda	3	13	-	-	-	390	336	86.2	390	336	86.2
Faridkot	3	5	10	9	90	140	131	93.6	150	140	93.3
Fazilka	2	8	44	33	75	196	155	79.1	240	188	78.3
Firozepur	1	1	-	-	-	30	29	96.7	30	29	96.7
Gurdaspur	1	1	-	-	-	30	7	23.3	30	7	23.3
Hoshiarpur	1	1	8	7	87.5	22	20	90.9	30	27	90
Jalandhar	1	1	-	-	-	30	27	90	30	27	90
Ludhiana	3	5	-	-	-	153	106	69.3	153	106	69.3
Mansa	2	3	23	23	100	67	12	17.9	90	35	38.9
Moga	1	1	4	1	25	26	16	61.5	30	17	56.7
Patiala	1	2	28	21	75	20	14	70	48	35	72.9
Rupnagar	1	1	-	-	-	30	6	20	30	6	20
Sangrur	5	8	7	4	57.1	233	194	83.3	240	198	82.5
Tarn Taran	1	2	30	0	0	30	1	3.3	60	1	1.7
Newanshahr (sbs Nagar)	2	2	-	-	-	60	55	91.7	60	55	91.7
S. A. S Nagar	1	1	30	30	100				30	30	100
Muktsar Sahib	3	9	26	14	53.8	238	182	76.5	264	196	74.2
Grand Total, Prevalence at 95% CI	34	66	210	142	67.6 CI 95%: (61 to 74)	1755	1298	74 CI 95%: (72 to 76)	1965	1440	73.3 CI 95%: (71 to 75)

Table S18. Details of the population immunity status PPR (% Seroprevalence) in small ruminants in the state of Tamil Nadu

Name of the Districts in Tamil Nadu	No. of Taluks in the State	No. of Village/ Epi-Units	Sheep			Goat			Total		
			No. of serum samples screened	No. of samples positive in ELISA	Prevalence (%)	No. of serum samples screened	No. of samples positive in ELISA	Prevalence (%)	No. of serum samples screened	No. of samples positive in ELISA	Prevalence (%)
Ariyalur	3	3	-	-	-	108	85	78.7	108	85	78.7
Coimbatore	3	3	-	-	-	108	91	84.3	108	91	84.3
Cuddalore	5	10	51	48	94.1	309	258	83.5	360	306	85
Dharmapuri	2	2	24	11	45.8	48	41	85.4	72	52	72.2
Dindigul	4	4	33	20	60.6	111	78	70.3	144	98	68.1
Erode	3	3	33	32	97	75	71	94.7	108	103	95.4
Kanchipuram	2	2	51	46	90.2	21	21	100	72	67	93.1
Karur	2	2	45	45	100	27	27	100	72	72	100
Krishnagiri	2	2	45	28	62.2	27	24	88.9	72	52	72.2
Madurai	3	3	22	16	72.7	86	71	82.6	108	87	80.6
Nagapattinam	5	5	-	-	-	180	133	73.9	180	133	73.9
Namakkal	5	7	27	19	70.4	222	182	82	249	201	80.7
Pudukkottai	4	5	54	42	77.8	125	93	74.4	179	135	75.4
Ramanathapuram	3	3	44	34	77.3	63	59	93.7	107	93	86.9
Salem	5	8	33	22	66.7	252	208	82.5	285	230	80.7
Sivaganga	1	1	36	32	88.9	-	-	-	36	32	88.9
Thanjavur	3	4	9	9	100	135	119	88.1	144	128	88.9
Thiruvallur	3	3	33	23	69.7	75	47	62.7	108	70	64.8
Thiruvarur	2	3	-	-	-	108	97	89.8	108	97	89.8
Tiruchirappalli	7	11	57	38	66.7	339	294	86.7	396	332	83.8
Tirunelveli	5	5	74	65	87.8	106	83	78.3	180	148	82.2
Tiruppur	3	3	99	56	56.6	6	5	83.3	105	61	58.1
Tiruvannamalai	5	6	81	64	79	135	129	95.6	216	193	89.4
Tuticorin	4	5	27	19	70.4	153	132	86.3	180	151	83.9
Villupuram	7	12	178	163	91.6	254	233	91.7	432	396	91.7
Virudhunagar	4	7	106	98	92.5	146	132	90.4	252	230	91.3
Kanyakumari	1	1	-	-	-	36	35	97.2	36	35	97.2
Grand Total, Prevalence at 95% CI	96	123	1162	930	80 CI 95%: (78 to 82)	3255	2748	84.4 CI 95%: (83 to 86)	4417	3678	83.3 CI 95%: (82 to 84)

Table S19. Details of the population immunity status PPR (% Seroprevalence) in small ruminants in the state of Telangana

Name of the Districts in Telangana	No. of Taluks in the State	No. of Village/ Epi-Units	No. of serum samples screened	No. of samples positive in ELISA	Prevalence (%)	No. of serum samples screened	No. of samples positive in ELISA	Prevalence (%)	No. of serum samples screened	No. of samples positive in ELISA	Prevalence (%)
			Sheep			Goat			Total		
Adilabad	7	9	302	257	85.1	91	77	84.6	393	334	85
Bhadradri Kothagudem	2	2	65	50	76.9	25	24	96	90	74	82.2
Hyderabad	1	1	-	-	-	42	41	97.6	42	41	97.6
Jangoan	5	6	183	170	92.9	87	84	96.6	270	254	94.1
Jayashankar Bhupalapally	9	9	274	263	96	128	124	96.9	402	387	96.3
Jogulamba Gadwal	1	1	36	31	86.1	9	9	100	45	40	88.9
Kamareddy	4	5	139	126	90.6	83	72	86.7	222	198	89.2
Karimnagar	1	2	60	53	88.3	30	23	76.7	90	76	84.4
Khammam	7	8	173	161	93.1	184	178	96.7	357	339	95
Mahabubabad	5	5	154	147	95.5	68	68	100	222	215	96.8
Mahbubnagar	5	5	179	150	83.8	46	44	95.7	225	194	86.2
Mancherial	4	4	-	-	-	180	176	97.8	180	176	97.8
Medak	7	7	9	9	100	306	286	93.5	315	295	93.7
Medchal Malkajgiri	2	2	90	57	63.3	-	-	-	90	57	63.3
Nagarkurnool	8	9	319	255	79.9	81	68	84	400	323	80.8
Nalgonda	7	8	249	192	77.1	110	80	72.7	359	272	75.8
Nirmal	2	2	-	-	-	90	85	94.4	90	85	94.4
Nizamabad	9	9	212	185	87.3	186	167	89.8	398	352	88.4
Peddapalli	5	5	159	135	84.9	63	42	66.7	222	177	79.7
Rajanna Sircilla	3	3	71	63	88.7	61	47	77	132	110	83.3
Rangareddi	8	10	409	277	67.7	40	21	52.5	449	298	66.4
Sangareddy	6	6	-	-	-	268	240	89.6	268	240	89.6
Siddipet	5	5	8	8	100	217	190	87.6	225	198	88
Suryapet	4	5	211	161	76.3	14	13	92.9	225	174	77.3
Vikarabad	6	9	73	58	79.5	318	266	83.6	391	324	82.9
Wanaparthy	5	6	216	195	90.3	52	39	75	268	234	87.3
Warangal Rural	5	7	211	190	90	104	97	93.3	315	287	91.1
Yadadri Bhuvanagiri	6	6	227	154	67.8	37	26	70.3	264	180	68.2
Jagtial	2	2	56	37	66.1	34	28	82.4	90	65	72.2
Kumuram Bheem Asif Abad	2	2	65	63	96.9	22	21	95.5	87	84	96.6
Grand Total, Prevalence at 95% CI	143	160	4150	3447	83.1 CI 95%: (82 to 84)	2976	2636	88.6 CI 95%: (87 to 90)	7126	6083	85.4 CI 95%: (85 to 86)

Table S20. Details of the population immunity status PPR (% Seroprevalence) in small ruminants in the state of Uttarakhand

Name of the Districts in Uttarakhand	No. of Taluks in the State	No. of Village/ Epi-Units	Sheep			Goat			Total		
			No. of serum samples screened	No. of samples positive in ELISA	Prevalence (%)	No. of serum samples screened	No. of samples positive in ELISA	Prevalence (%)	No. of serum samples screened	No. of samples positive in ELISA	Prevalence (%)
Almora	8	9	-	-	-	268	79	29.5	268	79	29.5
Bageshwar	2	4	16	16	100	104	92	88.5	120	108	90
Chamoli	5	5	72	57	79.2	78	62	79.5	150	119	79.3
Champawat	2	3	-	-	-	90	45	50	90	45	50
Haridwar	1	1	-	-	-	29	23	79.3	29	23	79.3
Nainital	4	4	-	-	-	119	85	71.4	119	85	71.4
Pauri Garhwal	4	4	-	-	-	119	76	63.9	119	76	63.9
Pithoragarh	9	9	33	9	27.3	237	141	59.5	270	150	55.6
Rudraprayag	1	1	16	4	25	14	3	21.4	30	7	23.3
Tehri Garhwal	5	6	6	3	50	170	93	54.7	176	96	54.5
Udham Singh Nagar	1	1	-	-	-	30	28	93.3	30	28	93.3
Uttarkashi	5	8	74	52	70.3	144	107	74.3	218	159	72.9
Dehradun	5	12	6	4	66.7	349	257	73.6	355	261	73.5
Grand Total, Prevalence at 95% CI	52	67	223	145	65 CI 95%: (59 to 71)	1751	1091	62.3 CI 95%: (60 to 65)	1974	1236	62.6 CI 95%: (60 to 65)

Table S21. Details of the population immunity status PPR (% Seroprevalence) in small ruminants in the state of West Bengal

Name of the Districts in West Bengal	No. of Taluks in the State	No. of Village/ Epi-Units	Sheep			Goat			Total		
			No. of serum samples screened	No. of samples positive in ELISA	Prevalence (%)	No. of serum samples screened	No. of samples positive in ELISA	Prevalence (%)	No. of serum samples screened	No. of samples positive in ELISA	Prevalence (%)
24 Paraganas North	6	6	14	7	50	148	101	68.2	162	108	66.7
24 Paraganas South	1	2	9	9	100	45	27	60	54	36	66.7
Bankura	10	13	27	21	77.8	324	217	67	351	238	67.8
Bardhaman	9	12	51	42	82.4	269	197	73.2	320	239	74.7
Birbhum	4	5	12	6	50	123	59	48	135	65	48.1
Darjeeling	1	1	-	-	-	26	23	88.5	26	23	88.5
Dinajpur Dakshin	4	5	-	-	-	133	52	39.1	133	52	39.1
Hooghly	1	2	-	-	-	48	48	100	48	48	100
Howrah	3	4	-	-	-	108	45	41.7	108	45	41.7
Jalpaiguri	1	3	-	-	-	72	56	77.8	72	56	77.8
Jhargram	2	4	3	3	100	104	89	85.6	107	92	86
Medinipur East	3	3	-	-	-	81	43	53.1	81	43	53.1
Medinipur West	8	10	6	3	50	263	172	65.4	269	175	65.1
Murshidabad	5	5	3	2	66.7	132	109	82.6	135	111	82.2
Nadia	5	6	3	-	-	159	92	57.9	162	92	56.8
Purulia	3	3	-	-	-	81	39	48.1	81	39	48.1
Grand Total Prevalence at 95% CI	66	84	128	93	72.7 CI 95%: (65 to 80)	2116	1369	64.7 CI 95%: (63 to 67)	2244	1462	65.2 CI 95%: (63 to 67)

Table S22: Details of the PPRV antibodies prevalence distribution (in ≤30%, 31-70% and >70% prevalence) in small ruminants after the Second Annual Mass Vaccination implemented under the National PPR Eradication Programme

Name of States/Union Territories	<30 Seroprevalence	31-70 % Seroprevalence	>70 % Seroprevalence	<30 Seroprevalence %	31-70 % Seroprevalence %	>70 % Seroprevalence %
	Epi Unit distribution			Epi Unit distribution %		
Andhra Pradesh	7	30	111	4.7	20.3	75
Assam	16	20	44	20	25	55
Bihar	34	15	58	31.8	14	54.2
Chandigarh	0	1	3	0	25	75
Chhattisgarh	6	19	66	6.6	20.9	72.5
Goa	10	18	26	18.5	33.3	48.1
Gujarat	7	16	52	9.3	21.3	69.3
Karnataka	6	34	89	4.7	26.4	69
Ladakh	12	24	18	22.2	44.4	33.3
Madhya Pradesh	33	22	19	44.6	29.7	25.7
Maharashtra	13	14	48	17.3	18.7	64
Meghalaya	16	3	24	10.8	2	16.2
Mizoram	25	13	16	46.3	24.1	29.6
Odisha	4	17	69	4.4	18.9	76.7
Puducherry	16	20	64	16	20	64
Punjab	8	15	43	12.1	22.7	65.2
Tamil Nadu	1	23	99	0.8	18.7	80.5
Telangana	0	23	125	0	15.5	84.5
Uttarakhand	16	17	34	23.9	25.4	50.7
West Bengal	12	26	46	14.3	30.9	54.8
**Grand Total**	**242**	**370**	**1054**	**14.5**	**22.2**	**63.3**

Table S23: Chi-squared results of PPRV antibody prevalence in small ruminants after the Second Annual Mass Vaccination implemented under the National PPR Eradication Programme

Variables	No. of Samples Screened	No. of Samples Positive	Prevalence (%)	$\chi 2$	p-Value
**Sheep**	14397	11256	78.2	410.24	<0.005
**Goat**	37736	26131	69.2		
**Male**	13062	8893	68.1	113.3	<0.005
**Female**	39071	28494	72.9		
**6–12 months**	17993	12739	70.8	13.71	<0.005
**1–2 Years**	17307	12431	71.8		
**Above 2 Years**	16833	12217	72.6		
**Non-descriptive/ Indigenous/local**	47678	34113	71.5	7.57	>0.05
**Cross breed**	4455	3274	73.5		
**Nomadic**	2089	1348	64.5	481.8	<0.005
**Intensive**	2529	1611	63.7		
**Semi-Intensive**	30534	21262	69.6		
**Extensive (Free range)**	16981	13166	77.5		
